# The anti‐*Trypanosoma* activities of medicinal plants: A systematic review of the literature

**DOI:** 10.1002/vms3.912

**Published:** 2022-08-29

**Authors:** Shahin Nekoei, Faham Khamesipour, Solomon Habtemariam, Wanderley de Souza, Pardis Mohammadi Pour, Seyed Reza Hosseini

**Affiliations:** ^1^ Faculty of Veterinary Medicine Shahrekord Branch Islamic Azad University Shahrekord Iran; ^2^ Center for Research and Training in Skin Diseases and Leprosy Tehran University of Medical Sciences Tehran Iran; ^3^ Pharmacognosy Research Laboratories and Herbal Analysis Services University of Greenwich Central Avenue Chatham‐Maritime Gillingham Kent UK; ^4^ Laboratório de Ultraestrutura Celular Hertha Meyer, Instituto de Biofísica Carlos Chagas Filho Universidade Federal do Rio de Janeiro Rio de Janeiro RJ Brazil; ^5^ Instituto Nacional de Ciência e Tecnologia em Biologia Estrutural e Bioimagens e Centro Nacional de Biologia Estrutural e Bioimagens Universidade Federal do Rio de Janeiro Rio de Janeiro RJ Brazil; ^6^ Phytochemistry Research Center Shahid Beheshti University of Medical Sciences Tehran Iran

**Keywords:** anti‐trypanosomal activity, medicinal plants, review

## Abstract

**Background:**

The existing drug treatments for trypanosomiases are limited and suffer from shortcomings due to their toxicity and the emergence of resistant parasites. Developing anti‐trypanosomal compounds based on natural products is a promising way of fighting trypanosomiases.

**Objectives:**

This study aims to identify through scientific review a large variety of medicinal plants (anti‐trypanosomal) used worldwide and scientifically shown to display anti‐trypanosomal effects.

**Methods:**

To collect data, the anti‐trypanosomal activities of Africa, Asia, the Middle East, South America, North America, Europe and Oceania medicinal plants have been checked by considering the published paper.

**Results:**

Based on collected data, 77 natural molecules were reported in the literature. Of which 59 were from the African region, 11 from Asia, 3 from Europe and 4 from Latin America. These active components belong to alkaloids, triterpenoids, lactone, quinoids, flavonoids, iridoids, lignans, steroids, lipids, oxygenated heterocycles, benzenoids, proteins, coumarins, phenylpropanoids and peptides. We also specified the prosperous plants with unique anti‐trypanosomal activities.

**Conclusions:**

However, there is a need for further studies on the ability of the isolated compounds to ameliorate the trypanosome‐induced pathological alterations and also the elucidation of their modes of actions and activities against other trypanosome species.

## 1 INTRODUCTION

Trypanosomiases are a widespread vector‐borne disease globally that affects humans and domestic and wild animals. The pathophysiology of these diseases may vary depending on the pathogenic species involved and the host. Its symptoms in humans include irregular fever and septicemia. At the same time, in animals, a decrease in the number of red blood cells and body mass can lead to unproductivity and death (Osório et al., 2008). Trypanosomiases have been considered a significant public health problem in animals and humans (Hassan et al., 2020).

The global prevalence of trypanosomiases, in general, is underreported (Wilkinson and Kelly, 2009). The two significant trypanosomiases in humans are the African Trypanosomiases (HAT, also known as sleeping sickness) and Chagas disease, caused by *Trypanosoma brucei* and *Trypanosoma cruzi*. According to the World Health Organization (PAHO, 2016) data, *T. cruzi* infects about 5–6 million people worldwide and causes approximately 10,000 deaths per year (WHO, 2015). For HAT, its incidence is now at a historic low, with fewer than 1000 cases reported in 2018 (WHO, 2018).

A small number of trypanocidal drugs have shown efficacy against the two species of parasites. These include two approved drugs that can treat Chagas Disease during its acute phase (Benznidazole and Nifurtimox) (Sepúlveda‐Robles et al., 2019). The recommended drugs to treat the HAT include suramin (EC: 205‐658‐4), pentamidine (EC: 205‐424‐1), melarsoprol (EC: 207‐793‐4) and Fexinidazole Winthrop (Dickie et al., 2020). Fexinidazole is a DNA synthesis inhibitor for the Neglected Diseases initiative (DNDi) for the oral treatment of HAT and Chagas’ disease, which shows activity against *Trypanosoma brucei gambiense* and *T. b. rhodesiense* as well as preceeds through Phase II clinical trial based on FDA definition (Deeks, 2019). The other drugs, including Nifurtimox, are in Phase III clinical trials, and Eflornithine (EC: 205‐658‐4) has not yet entered into clinical trial stages. However, the first three drugs have limitations, including poor efficacy, potential adverse effects and the development of resistance by the parasites (Wilkinson and Kelly, 2009). Oral fexinidazole is a valuable first‐line treatment option in the early stages of (stage 1 or early stage 2) African *Trypanosoma brucei gambiense* (Kande Betu Ku Mesu et al., 2021). Eflornithine is a standard treatment for second‐stage therapy, and nifurtimox‐eflornithine combination therapy is a proper combination for first‐line use in HAT control programs (Priotto et al., 2009). Additionally, DNDi has developed another oral therapy, acoziborole, suitable for the treatment of both stage 1 and stage 2 disease in a single dose (Dickie et al., 2020).

Also, the neglected disease status means a little economic benefit for developing novel drugs in this field (Dickie et al., 2020). There is little interest in developing drugs against these diseases because they are neglected. However, they are called ‘neglected diseases’ because pharmaceutical companies have little interest in investing in them, as fexinidazole has recently met that need for *T. brucei gambiense* (Kande Betu Ku Mesu et al., 2021). However, melarsoprol is very toxic and is still being used against *T. brucei rhodesiense* (Fairlamb and Horn, 2018), and resistance may still arise against fexinidazole, so new lead compounds for drugs against these parasites remain essential.  Thus, there has been a considerable need to find new trypanocidal agents with better efficacy and safety profiles.

Natural products are valuable sources for discovering and developing effective medicines against various diseases (Hashemi et al., 2021; Newman and Cragg, 2016; Nezaratizade et al., 2021; Tajbakhsh et al., 2021a; Tajbakhsh et al., 2021b). The WHO report highlighted that a quarter of currently useful drugs had been derived from traditional plants. For many parts of the world, especially where trypanosomiases are prevalent in Africa, India, China, the Middle East and South Asia, traditional medicines with local preparations are the predominant means of therapy (Ahmad Khan and Ahmad, 2019). These countries are also endowed with tremendous medicinal plant resources, some of which have shown efficacy under in vitro and/or in vivo conditions. At present, the available reviews in this field report anti‐trypanosomal activity for particular regions, such as the African region (Ibrahim et al., 2014; Lawal et al., 2015), Myanmar (Asia) (Bawm, 2010) and Saudi Arabia (Al‐Musayeib et al., 2012). These exciting but somehow dated but interesting publications reported a lot of medicinal plants and some isolated active compounds. Finally, the current, up‐to‐date review covers natural products isolated from plants used worldwide and active against trypanosomiases.

### 1.1 Ethnopharmacology of anti‐trypanosomal medicinal plants in Africa continent

Since the primitive period, herbs have been a valuable source of medication for both human and livestock diseases (Odhiambo et al., 2011). During these thousand years of observation, it has been found that different parts of herbs possess healing properties. With the advancement in pharmaceutical and medical sciences, phytoconstituents were subsequently confirmed to be accountable for the curative characteristics of plants. Nowadays, high‐tech methods have resulted in the isolation and elucidation of these phytoconstituents. Some of these phytoconstituents have served as lead compounds to develop chemotherapeutic drugs against diseases, whether infectious or non‐infectious (Kasilo et al., 2010).

On the one hand, some modern drugs have their ethnopharmacological sources. Nevertheless, despite technological advances, the discovery of new drugs faces a primary innovation deficit that unfavourably impacts the pharmaceutical industry. On the other hand, current studies demonstrate that entry barriers have decreased for introducing a new drug (DiMasi & Paquette, 2004; Patwardhan, 2005).  Seventy‐five per cent of the approved anti‐infectious disease drugs from 1981 to 2002 are natural origins (Newman et al., 2003), while 61% of all new chemical compounds presented as drugs during the same period could be considered natural products (Gupta et al., 2005).

Aside from this significant role of medicinal herbs in drug discovery, the use of local herbal products provides the only option for therapeutic purposes for African populations. The primary reason for this issue is the lack of a sound health care system in some parts of the continent, which causes the population's vulnerability to many infectious diseases (Elujoba et al., 2005). Eighty per cent of the African population depends almost entirely on herbal medicinal products for their primitive health care needs (Kasilo et al., 2010).

One of the significant infections that severely affect humans and animals in Africa is African trypanosomiasis, also called ‘sleeping sickness’ in humans or ‘Nagana’ in animals. (Atawodi 2005; Welburn et al., 2009). It is one of the most neglected parasitic diseases that affect human health and dramatically reduces Africa's livestock productivity (Atawodi 2005; Welburn et al., 2009). Preliminary estimates show that almost 70 million people distributed over 1.55 million km^2^ in Africa are at risk of this infectious disease (Simarro et al., 2012). In addition, animal trypanosomiases, or Nagana, are distributed over nearly 25 million km^2^ in Africa, where livestock productivity fell by 50%. The important species in this disease include *Trypanosoma vivax*, *Trypanosoma congolense*, *Trypanosoma evansi*  and  *Trypanosoma brucei* (Mbaya et al., 2009). Currently, the African trypanosomiases chemotherapy remains abandoned due to the available approved drugs with some concerns, including parasite resistance, toxicity, poor availability, high cost and parenteral root of administration (Ibrahim et al., 2014). Fortunately, the continent has vast resources of medicinal plants that are traditionally used to cure this disease. This is evident in the tendency to use ethnobotanical science to manage disease in different parts of Africa (Atawodi et al., 2002; Ntie‐Kang et al., 2013). It is important to note that studies have confirmed the impact of these African herbal remedies as anti‐trypanosomal agents under in vitro and/or in vivo models. Hence, a critical review of these studies (anti‐trypanosomal) African medicinal plants in the African continent and anti‐trypanosomal plants in other continents is required to provide a comprehensive record to specify gaps in knowledge about the basic strategies to address such gaps.

## 2 MATERIALS AND METHODS

### 2.1 Search strategy

Literature about medicinal plants (with anti‐trypanosomal activity) was collected online from published articles using the keywords: ‘Trypanosoma AND medicinal plant’, ‘Trypanosoma AND natural product’ from 1960 to May 2020. These keywords were entered into the primary scientific databases, such as PubMed, Science Direct, Scopus and Google scholar. The articles obtained were included based on the reliability of their source. Some articles were found by examining the bibliography of other publications or by directly accessing the webpage of the journal.

### 2.2 Inclusion and exclusion criteria

The documents used were selected based on several criteria: (a) they have published articles or doctoral theses, (b) research has been carried out on antiparasitic medicinal plants in general and anti‐trypanosomal plants in particular, (c) mention at least the minimum inhibitory concentration or the inhibition degree of the extract(s) or isolated compound(s) considering the anti‐trypanosomal activity, (d) in cases where different authors present results for the same plants, the most recent results are prioritised unless they present more minor details such as cytotoxicity tests, (e) due to the volume of data available for African region medicinal plants, only plants whose bioactive compound have been isolated were reported herein. The EC50 below 25 µM or µg for pure compounds was considered the search limit for the whole region. The author aimed to review the tested medicinal plant extracts, not just the isolated compounds from plants. Literature was not used when the results came from an ethnobotanical survey without scientific investigation.

### 2.3 Data extraction

The information such as the species and family of the plant, the type of extraction, the active compound(s) if isolated, the strain of Trypanosoma tested, the 50% effective concentration and cytotoxic concentration, country of study and the name of the author were extracted from relevant literature and presented in the form of a table according to geographical location.

### 2.4 Ethical approval statement

An ethics statement is not applicable because this study is based exclusively on published literature.

## 3 RESULTS AND DISCUSSION

### 3.1 Analysis of the included literature

**FIGURE 1:**
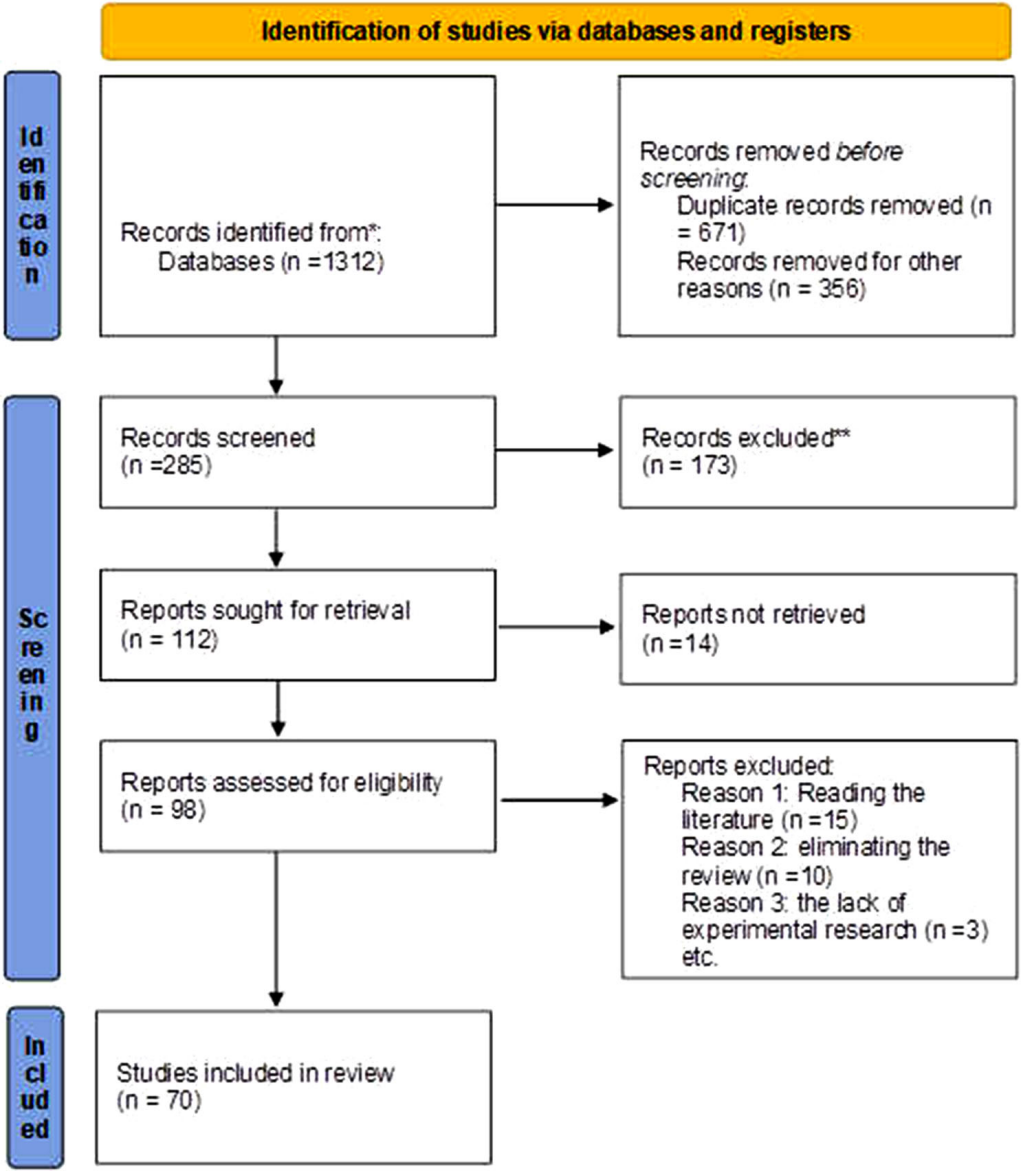
PRISMA 2020 flow diagram for which included searches of databases

A total of 70 articles have been selected based on the inclusion criteria. Twenty studies reported African anti‐trypanosomal plants, 11 reported Asian anti‐trypanosomal plants, three reported the Middle East anti‐trypanosomal plants and 15 reported Latin American anti‐trypanosomal plants. Two studies reported North American anti‐trypanosomal plants, and nine studies reported European anti‐trypanosomal plants. One study reported Oceania's anti‐trypanosomal plants (Figure 1). A total of 70 relevant kinds of literature have been selected based on the inclusion criteria. The PRISMA 2020 flow diagram shows 25, 16, 18, 1, 9 and 1, including database searches (Figure 2) (Page et al., 2021).

FIGURE 2Chemical structures of isolated compounds from Africa medicinal plants

**Figure:**
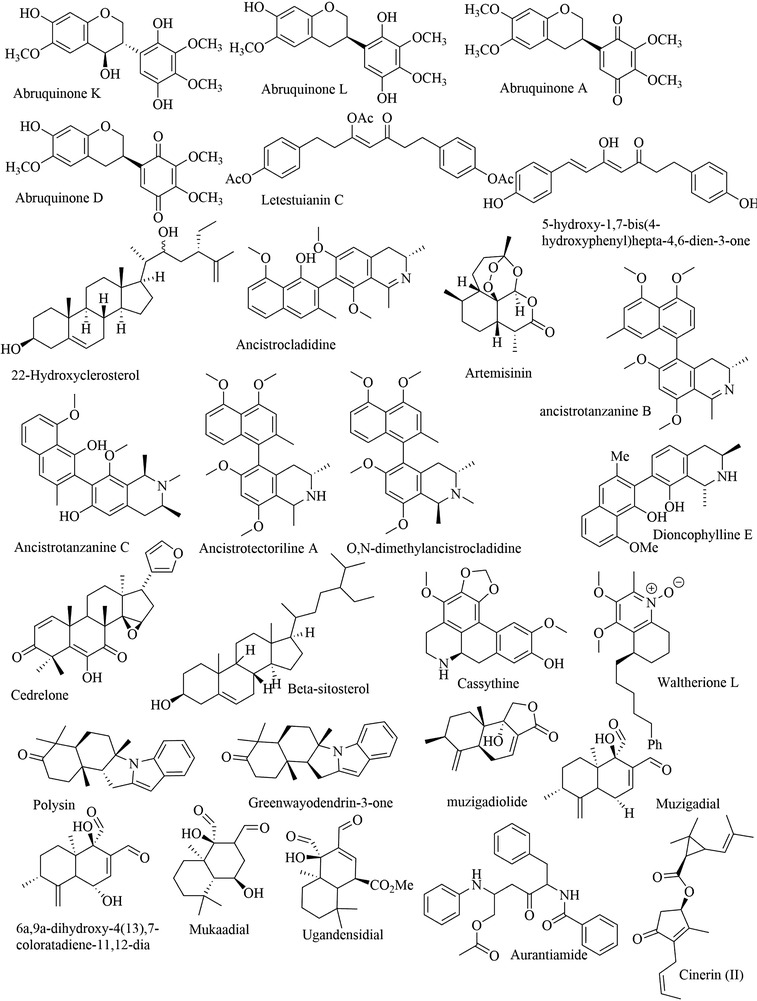

**Figure:**
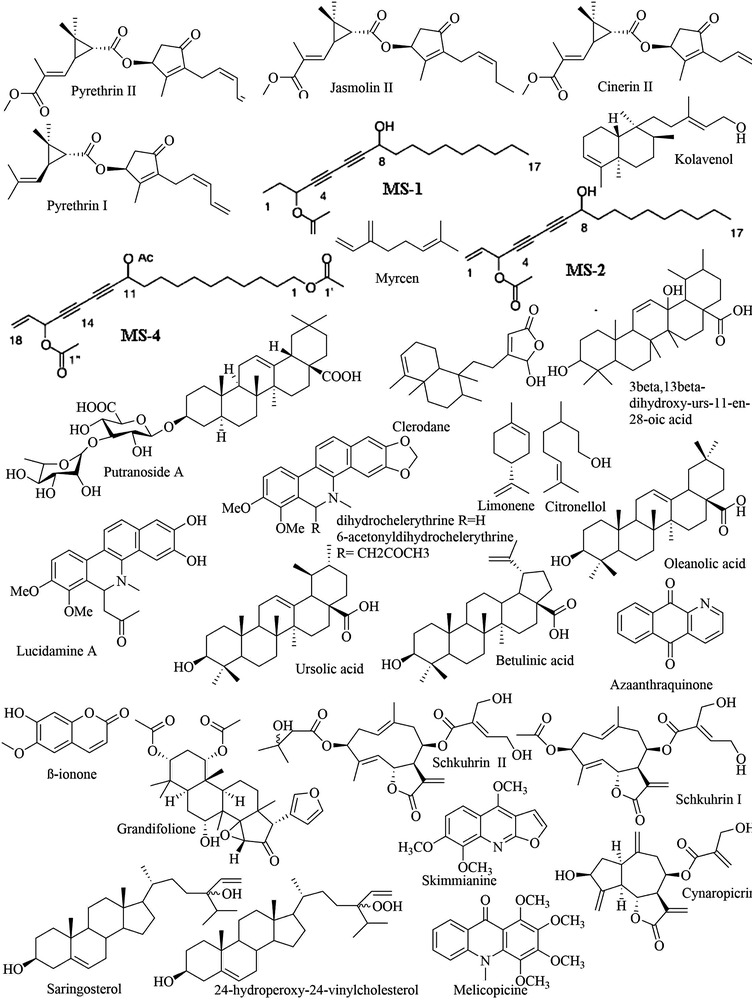

**Figure:**
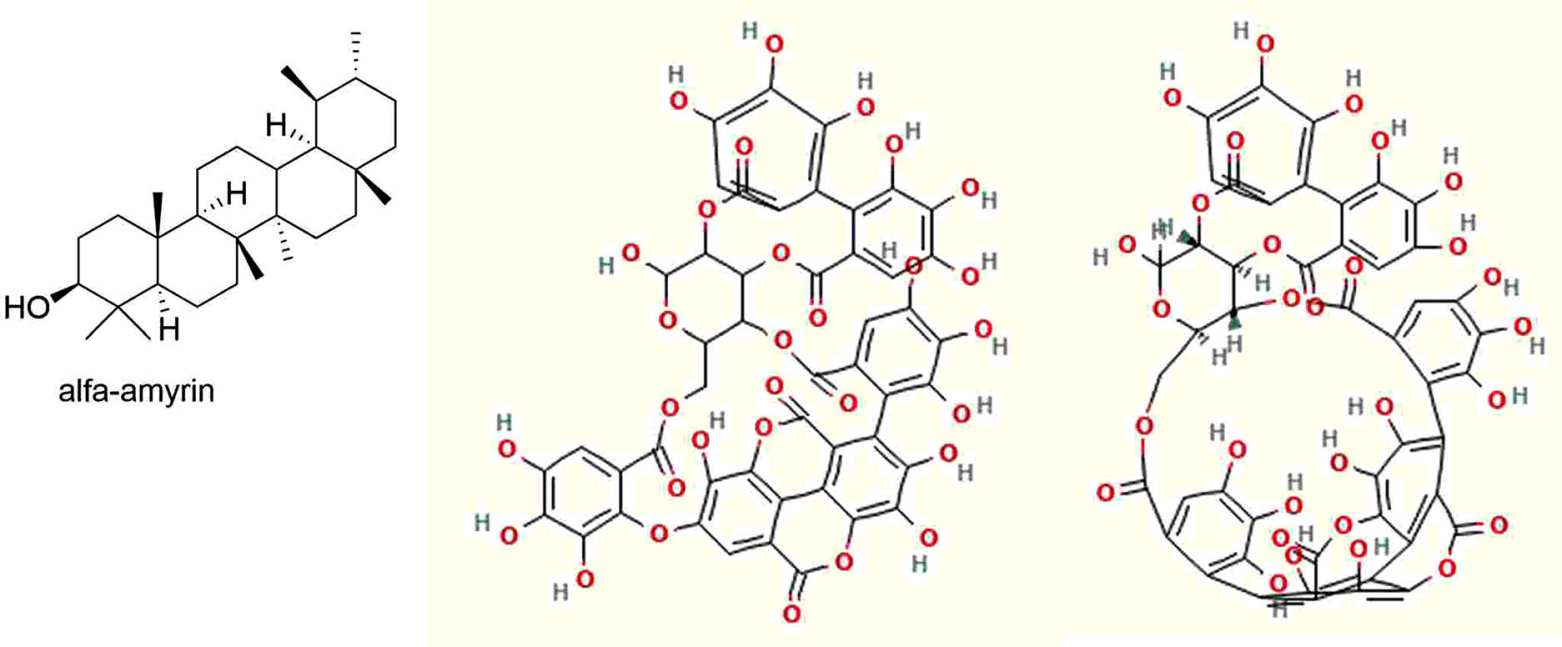

### 3.2 African region plants

**FIGURE 3:**
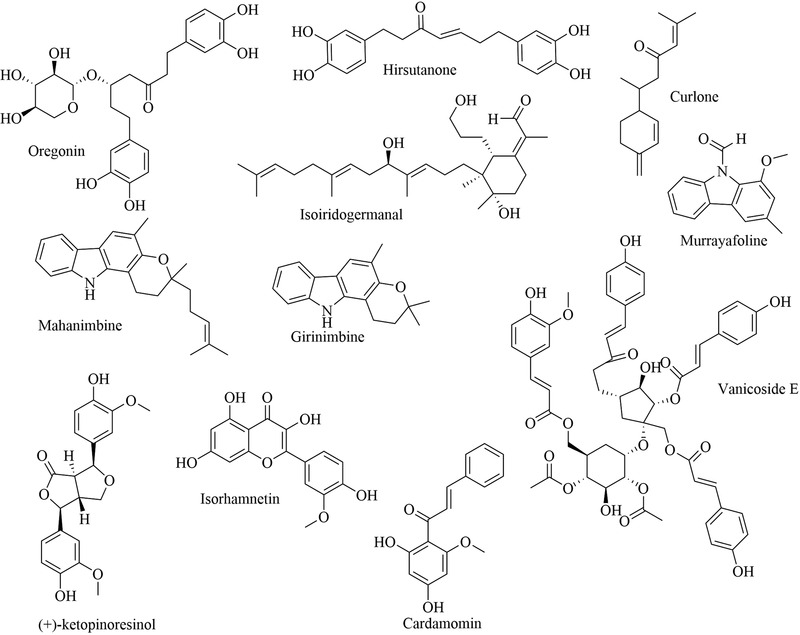
Chemical structures of isolated compounds from Asia medicinal plants

Ethnobotanical resources for Africa demonstrated unusual plants with anti‐trypanosomal activity (Ibrahim et al., 2014; Lawal et al., 2015). We explained 264 and 215 plants, respectively, which were assessed for anti‐trypanosomal activity. Due to the high amount of data available for African region anti‐trypanosomal plants, only the plants with the minimum inhibitory concentration of the bioactive compound were scrutinised (Table 1 and Figure 3).

**TABLE 1:** Plants assessed for anti‐trypanosomal activity and the EC50 of bioactive compounds isolated

**Scientific name**	**Family**	Part(s) used	**Solvent**	Bioactive compound(s)	**Model**	Sub species	**cytotoxic/ biological activity**	**EC50**	**Country**	References
*Abrus precatorius L. subsp. africanus Verdc*	Leguminosae	Leaf	Methanol	Abruquinone K, L, A and D	In vitro	*T. b. r*	57.3, 7.5, 34.5 and 4.8 µM	0.1, 0.02, 0.02 and 0.01 µM	South Africa	(Hata et al., 2014)
*Aframomum letestuanum Gagnep*	Zingiberaceae	Seed	DCM	Letestuianin C and 5‐hydroxy‐1,7‐bis(4‐hydroxyphenyl)hepta‐4,6‐dien‐3‐one	In vitro	*T.b. b*	‐	1.4 and 2.6 µg/ml	Cameroon	(Kamnaing et al., 2003)
*Allexis cauliflora (Oliv.) Pierre*	Violaceae	Leaf	DCM	22‐Hydroxyclerosterol	In vitro	*T.b. b*	1.12 µM	1.56 µM inhibit the glycolytic enzyme PGI	Cameroon	(Nganso et al., 2011)
*Ancistrocladus abbreviatus subsp. lateralis Gereau*	Ancistrocladaceae	Leaves, stem bark and roots	DCM	Ancistrocladidine, Ancistrotanzanines B and C, Ancistrotectoriline A and O,N‐dimethylancistrocladidine	In vitro	*T.b.r* *T.c*	28.3, 8.1, 40.7, 6.5 and 42.9 µg/ml	0.17 to 12.41 µM	Cameroon	(Simoben et al., 2018) (Bringmann et al. 2003, Bringmann et al. 2004)
*Solanecio angulatus* *Crotalaria phillipsiae* *Artemisia annua L*.	Fabaceae Asteraceae	Flower Twigs Leaf	DCM/ methanol	Artemisinin	In vitro	*T.b. b*	>500 27.39	12.17^e^, 12.47^e^ µg/ml and 0.127 µM	Tanzania	(Nibret et al., 2009)
*Azadirachta indica A.Juss*.	Meliaceae	Leaf	Chloroform	7,15‐dihydroxy‐7,15‐deoxo nimbin	In vitro	*T.b. r*		15.6 µg/ml	Kenya	(Githua and Hassanali, 2011)
*Buchholzia coriacea Engl*	Capparaceae	Seeds	Methanol	Beta‐sitosterol α–sulphur	In vitro	*T.b. b*	No noticeable morphological changes	12.5 and 25 µg/ml	Nigeria	(Nweze, Anene and Asuzu, 2011)
*Cassytha filiformis L*.	Lauraceae	Leaf	DCM	Cassythine	In vitro	*T.b. b*	15.2 µM	6 µM	Cameroon	(Simoben et al., 2018)
*Chrysanthemum cinerariifolium (Trevir.) Vis*	Asteraceae	Flowers	n‐hexane	Cinerin (II) Pyrethrin (I,II) Jasmolin (II)	In vitro	*T.b. r*	28, 146.6, 95.1 and 31.5	12.2 6.9, 10.6 12 µg/ml	South Africa	(Hata et al., 2011)
*Cussonia zimmermannii Harms*	Araliaceae	Root bark	Petroleum ether extract	Polyacetylenes (MS‐1, MS‐2 and MS‐4)	In vitro	*T.b.r/T.cr*	54(17), 12(3.6) and 58(22) µM (µg/ml)	18 (5.4), 0.46 (0.14) and 1.1 (0.42)/ 26 (7.9), 0.65 (0.20) and 0.40 (0.15) µM (µg/ml)	Tanzania	(Senn et al., 2007)
*Dioncophyllum thollonii Baill*	Dioncophyllaceae	Roots	DCM	Dioncophylline E	In vitro	*T.b.r/T.cr*	‐	0.73 and 18.4 µg/ml	Cameroon	(Simoben et al., 2018)
*Drypetes gerrardii Hutch*.	Putranjivaceae	Stem	DCM/methanol	Putranoside A	In vitro	*T.b. r*	68.2 µM	18.0 µM	South Africa	(Hata et al., 2014)
*Entada abyssinica A.Rich*.	Leguminosae	Stem	Ethanol	Kolavenol	In vitro	*T.b. r*	‐	2.5 mg/ml (8.6 mM)	Tanzania	(Freiburghaus et al., 1998)
*Eucalyptus maculata Hook*.	Myrtaceae	Leaf	Hexane, ethyl acetate and methanol	Triterpenoid (3β,13β‐dihydroxy‐urs‐11‐en‐28‐oic acid)	In vitro	*T. strains s427 WT, B48* and *AQP2/3KO*	1.58 µg/ml	1.58, 1.55 and 1.39 µg/ml	Nigeria	(Ebiloma et al., 2017)
*Garcinia lucida Vesque*	Clusiacea	Stem	DCM	Dihydrochelerythrine, 6‐acetonyldihydrochelerythrine, Lucidamine A	In vitro	*T.b. b*	35.4, 15.3 and 11.6 µM	0.8, 3.9 and 14.1 µM	Cameroon	(Fotie et al., 2007)
*Keetia leucantha (K.Krause) Bridson*	Rubiaceae	Leaf	DCM	Oleanolic acid/ursolic acid/betulinic acid/β‐ionone	In vitro	*T. b.b*	‐	7.3, 2.5, 19.1, 10.5 µg/ml	Nigeria	(Bero et al., 2013)
*Khaya anthotheca (Welw.) C.DC*.	Meliaceae	Seeds	Pet. ether	Grandifolione	In vitro	*T.b.r/T.cr*	44.7	10.66/20.9 µg/ml	Uganda	(Oboh, Lawal and Malann, 2013)
*Mitracarpus scaber Zucc. ex Schult. & Schult.f*.	Rubiaceae	Leaf	Methanol	Azaanthra‐quinone	In vitro/in vivo	*T.co* in bloodstream of BalbC mice, 50 mg/kg/d	‐/Inhibit reduced coenzyme Q_1_‐dependent O_2_	50 µg/ml	Nigeria	(Nok, 2002)
*Morinda lucida Benth*.	Rubiaceae	Leaves	Methanol	β‐sitosterol	In vitro	*T.b. b*	100	12.5 µg/ml	Nigeria	(Nweze, 2012)
*Ocimum gratissimum L*.	Lamilaceae	Seed oil	Oil	Myrcen, Limonen and Citronellal	In vitro	*T.b. b*	>50, >50 and >50 µg/ml	2.24, 4.24 and 2.76 µg/ml	Benin	(Kpadonou Kpoviessi et al., 2014)
*Polyalthia longifolia (Sonn.) Thwaites*	Annonaceae	Leaf	Hexane, ethyl acetate and methanol	Clerodane	In vitro	*T.co*	‐	0.38 µg/ml	Nigeria	(Ebiloma et al., 2017)
*Polyalthia suaveolens Engl. & Diels*	Annonaceae	Leaf	DCM	Mixture of polysin and greenwayodendrin‐3‐one	In vitro	*T.b.b*	170 µM	18 µM	Cameroon	(Simoben et al., 2018)
*Schkuhria pinnata (Lam.) Kuntze ex Thell*.	Asteraceae	Whole plant	DCM/methanol	Schkuhrin I and II	In vitro	*T.b. r/T.cr*	5.26 and 9.03 µM	0.9 and 1.5 µM/16.4 and 26.9 µM	South Africa	(Mokoka et al., 2013)
*Strychnos spinosa Lam*.	Loganiaceae	Leaf	Ipophilic	Saringosterol, 24‐hydroperoxy‐24‐vinylcholesterol	In vitro	*T.b.b*	>233.3 and 16.4 µM	7.8 and 3.2 µM	Tanzania	(Hoet et al., 2007)
*Teclea trichocarpa (Engl.) Engl*.	Rutaceae	Leaves	Methanol	Melicopicine, skimmianine and α‐amyrin	In vitro	*T.b.r*	>90, 38.6 and >90 µg/ml	15.56, 15.78, 11.21 µg/ml	Kenya	(Mwangi et al., 2010)
*Terminalia actinophylla Mart*.	Combretaceae	Leaf	Water	Terchebulin and punicalagin	In vitro	*T.b. b*	≥1500 and ≥1500 µg/ml	25 and 14 µM	Nigeria	(Shuaibu et al., 2008)
*Toona ciliata M.Roem*.	Meliaceae	Root	Methanol chloroform	Cedrelone	In vitro	*T.b. r*	‐	6.95^Me^, 3.2^Ce^ and 7.85	Kenya	(Githua and Hassanali, 2011)
*Vernonia guineensis Benth*.	Asteraceae	Stem bark	Ethanol	Vernoguinosterol and vernoguinoside	In vitro	*T.b. r*	‐	3–5 µg/ml	Cameroon	(Tchinda et al., 2002)
*Vernonia mespilifolia Less*.	Asteraceae	Leaf	DCM/methanol	Cynaropicrin	In vitro	*T.b. r/T.cr*	1.29 µM	0.23 µM/5.14 µM	South Africa	(Mokoka et al., 2013)
*Waltheria indica L*.	Malvaceae	Root	DCM	Waltheriones L	In vitro	*T.cr/ T.b.b/T.b.r*	‐	0.74^e^, 20^e^, 17.4^e^ µg/ml and 3.1 µM	Cameroon	(Simoben et al., 2018)
*Warburgia ugandensis subsp. ugandensis*	Canellaceae	Leaf	DCM	Muzigadiolide muzigadial, 6α,9α‐dihydroxy‐4(13),7‐coloratadiene‐11,12‐dial and mukaadial and ugandensidial	In vitro	*T.b.r*	‐	0.64 to 6.4 µM	Cameroon	(Simoben et al., 2018)
*Zapoteca portoricensis (Jacq.) H.M.Hern*.	Fabaceae	Leaf	DCM	Saropeptide or aurantiamide acetate	In vitro	*T.b.r/T.c*	92.05 µM	3.63 and 41.65 µM	Cameroon	(Simoben et al., 2018)

Abbreviations: EC50, half maximal inhibitory concentration (µg/ml); *T.b. b, Trypanosoma brucei brucei*; *T.e, Trypanosoma evansi*; *T. co, Trypanosoma congolense*

^e^Extract.

^Me^Methanolic extract.

^Ce^Chloroform extract.

More than 200 investigated plants (Ibrahim et al., 2014; Lawal et al., 2015) show potential trypanocidal activity; only 34 plants have their active compounds isolated in pure form. Only their compounds (flavonoid, saponins, alkaloid etc.) are reported for the other plants. This is due to the lack of resources in Africa to isolate the active molecules. Among these 34 plants, just six have been investigated in vivo. The anti‐trypanosomal activity of the extracts was most assessed on *Trypanosoma brucei* subspecies, which are responsible for African trypanosomiases (WHO, 2015). Considering the importance of trypanosomiases caused by this species in Africa, the development of anti‐trypanosomal medicine based on plants has been an exciting research topic.

Additionally, the medicinal plants of Africa provide a large variety of bioactive compounds. Of the 24 plants reported in Table 1, approximately 34 different bioactive compounds were isolated with trypanosomiases activity. Some plants, such as *Chrysanthemum cinerariifolium, Keetia leucantha, Tecla trichocarpa* and *Terminalia avicenioides*, provide at least three different potent bioactive compounds.

Concerning the criteria for choosing compounds with anti‐trypanosomal potential, EC50>20 µg/ml is considered ineffective (Pink et al., 2005). Thus, approximately 40 compounds seem to be effective (EC50<20 µg/ml) and have demonstrated promising anti‐trypanocidal activity (Figure 2). Given the minimum inhibitory concentration, only abruquinone (0.01 µg/ml) has a concentration closer to Melarsoprol reference (0.004 µg/ml) was active against *Trypanosoma brucei* and benznidazole (0.482 µg/ml) reference was active against *Trypanosoma cruzi*.

### 3.3 Asia plants

Asia plants assessed for anti‐trypanosomal activity with EC50 values for inhibition of parasites and cytotoxicity are shown in Table 2.

**TABLE 2:** Plants assessed for anti‐trypanosomal activity in in vitro model

Scientific name	Family	Part (s) used	Solvent	Bioactive compound	Sub species	EC50	CC_50_ *	Country	References
*Alnus japonica* (Thunb.) Steud.	Betulaceae	Bark	DCM	Oregonin Hirsutanone	*T.b*	1.14 and 1.78 µM	50 µM	Japan	(Tung et al., 2014)
*Aquilaria malaccensis Lam*.	Thymelaeaceae	Leaves	Ethanol	‐	*T.e*	128.63 µg/ml	259.78 µg/ml	Malaysia	(Dyary et al., 2014)
*Andrographis paniculata (Burm.f.) Nees*	*Acanthaceae*	Leaves/stems	Methanol	‐	*T.e*	54.7 µg/ml	55.1 µg/ml	Japan	(Bawm, 2010)
*Brucea javanica (L.) Merr*.	*Simaroubaceae*	Fruit	Methanol	‐	*T.e* *T.b*	27.2 µg/ml	309.15 µg/ml	Japan	(Bawm, 2010)
*Combretum acuminatum Roxb*.	*Combretaceae*	Rhizomes	Methanol	‐	*T.e*	90.7 µg/ml	853.15 µg/ml	Japan	(Bawm, 2010)
*Cordyline terminalis (L.) Kunth*	*Liliaceae*	Leaves	Water	‐	*T.e*	48.1 µg/ml		Malaysia	(Dyary et al., 2019)
*Crateva religiosa G.Forst*	*Capparidaceae*	Leaves/stems	Methanol	‐	*T.e*	107.1 µg/ml	691 µg/ml	Japan	(Bawm, 2010)
*Curcuma longa L*.	*Zingiberaceae*	Leaves	Oil	Curlone	*T.b.b*	1.38 µg/ml		Vietnam	(Le et al., 2019)
*Curcuma zedoaria (Christm.) Roscoe*	*Zingiberaceae*	Leaves	Oil	‐	*T.b.b*	2.51 µg/ml		Vietnam	(Le et al., 2019)
*Derris elliptica (Wall.) Benth*.	*Fabaceae*	Leaves	Ethanolic	‐	*T.e*	17.79 µg/ml	331.90 µg/ml	Malaysia	(Dyary et al., 2014)
*Eucalyptus globulus Labill*.	*Myrtaceae*	Leaf	Methanol	‐	*T.e*	51.1 µg/ml	622.95 µg/ml	Japan	(Bawm, 2010)
*Garcinia hombroniana Pierre*	*Clusiaceae*	Leaves	Ethanolic	‐	*T.e*	103.44 µg/ml	10.17 µg/ml	Malaysia	(Dyary et al., 2014)
*Goniothalamus tapis Miq*.	*Annonaceae*	Leaves	Ethanolic	‐	*T.e*	7.61 µg/ml	‐	Malaysia	(Dyary et al., 2019)
*Goniothalamus umbrosus J.Sinclair*	*Annonaceae*	Leaves	Ethanolic	‐	*T.e*	2.30 µg/ml	29.10 µg/ml	Malaysia	(Dyary et al., 2014)
*Iris domestica (L.) Goldblatt & Mabb*.	*Iridaceae*	Leaves	Petroleum ether	Isoiridogermanal	*T.b.b*	3.60 µg/ml	136.00 µg/ml	China	(Pathiranage et al., 2016)
*Jatropha podagrica Hook*.	*Euphorbiaceae*	Fruit	Methanol	‐	*T.e*	52.3 µg/ml	652.7 µg/ml	Japan	(Bawm, 2010)
*Litsea cubeba (Lour.) Pers*.	*Lauraceae*	Leaves	Oil	‐	*T.b.b*	1.12 nL/ml		Vietnam	(Le et al., 2019)
*Murraya koenigii (L.) Spreng*.	*Rutaceae*	Leaves		Mahanimbine, murrayafoline and girinimbine	*T.e*	3.13, 6.35 and 10.16 µg/ml	745.58 µg/ml	Malaysia	(Dyary et al., 2019)
*Nigella sativa L*.	*Ranunculaceae*	Seeds	Ethanolic	‐	*T.e*	291.72 µg/ml	381.59 µg/ml	Malaysia	(Dyary et al., 2014)
*Orthosiphon stamineus Benth*.	*Labiatae*	Leaves	Methanol	‐	*T.e*	144.7 µg/ml	628.9 µg/ml	Japan	(Bawm, 2010)
*Phyllanthus simplex Retz*.	*Euphorbiaceae*	Leaves/stem	Methanol	‐	*T.e*	96.1 µg/ml	98.8 µg/ml	Japan	(Bawm, 2010)
*Plumbago rosea L*.	*Plumbaginaceae*	Flowers	Methanol	‐	*T.e*	156.7 µg/ml	557.05 µg/ml	Japan	(Bawm, 2010)
*Polygonum hydropiper L*.	*Polygonaceae*	Leaves	DCM	Vanicoside E, (+)‐ketopinoresinol, isorhamnetin and cardamomin	*T.b*	0.49–7.77 µg/ml	‐	China	(Xiao et al., 2017)
*Punica granatum L*.	*Lythraceae*	Leaves	Ethanol	‐	*T.e*	20 mg/ml	‐	India	(Kumar et al., 2014)
*Quercus borealis F.Michx*.	*‎Fagaceae*	Roots	Methanol	‐	*T.e*	250 µg/ml		India	(Shaba et al., 2011)
*Rhoeo discolor (L'Hér.) Hance*	*Commelinaceae*	Leaves	Methanol	‐	*T.e*	75.8 µg/ml	424.9 µg/ml	Japan	(Bawm, 2010)
*Scutellaria baicalensis Georgi*	*Lamiaceae*	Leaves	Water/chloroform	‐	*T.b*	11.43 µg/ml 19.56 µg/ml	‐	China	(Floyd, 2013)
*Strobilanthes abbreviata Y.F. Deng & J.R.I. Wood*	*Acanthaceae*	Leaves	Ethanolic	‐	*T.e*	52.54 µg/ml	355.21 µg/ml	Malaysia	(Dyary et al., 2014)
*Vitex arborea Desf*.	*Verbenaceae*	Leaves/stem	Methanol	‐	*T.e*	48.6 µg/ml	735.15 µg/ml	Japan	(Bawm, 2010)
*Vitis repens (Lam.) Wight & Arn*.	*Vitaceae*	Root bark	Methanol	‐	*T.e*	8.6 µg/ml	209.9 µg/ml	Japan	(Bawm, 2010)
*Zingiber officinale Roscoe*	*Zingiberaceae*	Leaves	Oil	‐	*T.b.b*	3.10 nL/ml		Vietnam	(Le et al., 2019)

*Note*: The EC50 values for inhibition of parasites and the cytotoxicity are shown

* The extract concentration that reduced the cell viability by 50% when compared to untreated controls.

A total of 31 plants with their minimum inhibitory concentration have been identified in the literature. Four plants (*V. repens, P. simplex, V. arborea* and *A. brevipedunculata*) already have bioactive compounds. These seven compounds include resveratrol (EC50 = 31.4), 11‐O‐acetyl‐bergenin (EC50 = 61.2), stigmas‐4‐ en‐3‐ one (EC50 = 62.8), lupeol (EC50 = 98.4), Ψ‐taraxasterone (EC50 = 115.4), hopenyl‐3β‐O‐palmitate (EC50 = 68.2) and β‐amyrin palmitate (EC50 = 60.8) (Bawm, 2010). The extracts were mostly evaluated on *Trypanosoma evansi* due to its prevalence in Asia (Dyary et al., 2014). Considering the potency criteria asserted by Pink et al. (2005), it was expressed that the isolated compounds with an EC50> 20 µg/ml were not considered effective drugs. Thus, the seven isolated compounds may not be considered lead drugs. There is a need to pursue investigations that isolate more effective compounds. On the other hand, as suggested by Pink et al. (2005), crude extracts with potent in vivo anti‐trypanosomal activity such as <100 mg/kg with no toxic effect below 800 mg/kg may be considered promising lead structures. None of the plants with in vitro data were evaluated in vivo. The minimum inhibitory concentration of the methanolic extract of *Goniothalamus umbrosus* (2.30 µg/ml) was the only extract with an activity profile closer to the diminazene aceturate (0.01140 µg/ml) reference against *Trypanosoma evansi*.

### 3.4 Middle East

Table 3 shows a list of plants in the Middle East with cytotoxicity values against trypanosome parasite activity. Al‐Musayeib et al. (2012) reported 41 medicinal plants used in Saudi Arabia that showed anti‐trypanosomal activity in vitro. All of their inhibitory activities are explained by the EC50 and CC50. However, no details have been given about their bioactive compounds except their secondary metabolite composition. Their activity profile in in vivo studies is unknown, so their therapeutic potential remains to be established.

**TABLE 3:** Plants assessed for in vitro model of anti‐trypanosomal activity in Saudi Arabia which the EC50 and the cytotoxicity values are known

ScientificName	Family	Part (s) used	Solvent	Sub species	EC50	CC_50_	References
*Ajuga bracteosa Wall. ex Benth*.	Labiatae	Leaves	Methanol	*T.c* *T.b.b*	28.8 μg/ml	31.2 μg/ml	(Al‐Musayeib, Mothana, Matheeussen, et al., 2012)
*Albizia lebbeck (L.) Benth*.	Leguminosae	Stems	Methanol	*T.c* *T.b.b*	8.7 μg/ml 8.1 μg/ml	32.0 μg/ml	(Al‐Musayeib, Mothana, Al‐Massarani, et al., 2012)
*Cadaba farinosa subsp. adenotricha (Gilg & Benedict) R.A.Graham*	Capparaceae	Leaves/stems	Methanol	*T.c* *T.b.b*	28.6 μg/ml 10.6 μg/ml	32.9 μg/ml	(Al‐Musayeib, Mothana, Al‐Massarani, et al., 2012)
*Cadaba glandulosa Forssk*.	Capparaceae	Leaves/Stems	Methanol	*T.c* *T.b.b*	36.5 μg/ml 16.4 μg/ml	>64.0 μg/ml	(Al‐Musayeib, Mothana, Al‐Massarani, et al., 2012)
*Caralluma quadrangula (Forssk.) N.E.Br*.	Asclepiad‐aceae	Leaves	Methanol	*T.c* *T.b.b*	>64.0 μg/ml 32.5 μg/ml	>64.0 μg/ml	(Al‐Musayeib, Mothana, Al‐Massarani, et al., 2012)
*Caralluma sinaica (Decne.) A.Berger*	Asclepiad‐aceae	Leaves	Methanol	*T.c* *T.b.b*	7.3 μg/ml 7.7 μg/ml	20.5 μg/ml	(Al‐Musayeib, Mothana, Al‐Massarani, et al., 2012)
*Celtis africana Burm.f*.	Cannabaceae	Leaves/stems	Methanol	*T.c* *T.b.b*	29.4 μg/ml >64.0 μg/ml	>64.0 μg/ml	(Al‐Musayeib, Mothana, Al‐Massarani, et al., 2012)
*Centaurea pseudosinaica Czerep*.	Asteraceae	Leaves	Methanol	*T.c* *T.b.b*	31.0 μg/ml 9.1 μg/ml	16.0 μg/ml	(Al‐Musayeib, Mothana, Al‐Massarani, et al., 2012)
*Chrozophora oblongifolia (Delile) A.Juss. ex Spreng*.	Euphorbiacea	Leaves	Methanol	*T.c* *T.b.b*	32.0 μg/ml 10.8 μg/ml	>64.0 μg/ml	(Al‐Musayeib, Mothana, Al‐Massarani, et al., 2012)
*Conocarpus lancifolius Engl*.	Combretaceae	Fruits	Methanol	*T.c* *T.b.b*	32.2 μg/ml 35.2 μg/ml	7.2 μg/ml	(Al‐Musayeib, Mothana, Al‐Massarani, et al., 2012)
*Cordia sinensis Lam*.	Boragin‐aceae	Leaves/stems	Methanol	*T.c* *T.b.b*	33.9 μg/ml 32.0 μg/ml	>64.0 μg/ml	(Al‐Musayeib, Mothana, Al‐Massarani, et al., 2012)
*Costus arabicus L*.	Zingiberaceae	Roots	Methanol	*T.c* *T.b.b*	13.8 μg/ml 30.0 μg/ml	38.5 μg/ml	(Al‐Musayeib, Mothana, Al‐Massarani, et al., 2012)
*Cupressus sempervirens L*.	Cupressaceae	Leaves	Methanol	*T.c* *T.b.b*	8.3 μg/ml 2.1 μg/ml	10.7 μg/ml	(Al‐Musayeib, Mothana, Al‐Massarani, et al., 2012)
*Dorstenia barnimiana Schweinf*.	Moraceae	Leaves	Methanol	*T.c* *T.b.b*	29.6 μg/ml 22.6 μg/ml	49.4 μg/ml	(Al‐Musayeib, Mothana, Al‐Massarani, et al., 2012)
*Dodonaea viscosa (L.) Jacq*.	Sapindaceae	Leaves	Methanol	*T.c* *T.b.b*	>64.0 μg/ml 11.1 μg/ml	>64.0 μg/ml	(Al‐Musayeib, Mothana, Al‐Massarani, et al., 2012)
*Enicostemma verticillare L*.	Gentianaceae	Leaves	Methanol	*T.c* *T.b.b*	>64.0 μg/ml	9.9 ± 1.1 μg/ml	(Al‐Musayeib, Mothana, Al‐Massarani, et al., 2012)
*Ficus cordata subsp. salicifolia (Vahl) C.C.Berg*	Moraceae	Leaves	Methanol	*T.c* *T.b.b*	26.3 μg/ml	8.2 μg/ml	(Al‐Musayeib, Mothana, Al‐Massarani, et al., 2012)
*Ficus ingens (Miq.) Miq*.	Moraceae	Leaves	Methanol	*T.c* *T.b.b*	31.2 μg/ml 8.0 μg/ml	32.5 μg/ml	(Al‐Musayeib, Mothana, Al‐Massarani, et al., 2012)
*Ficus palmata subsp. virgata Browicz*	Moraceae	Leaves	Methanol	*T.c* *T.b.b*	22.6 μg/ml 8.1 μg/ml	37.7 μg/ml	(Al‐Musayeib, Mothana, Al‐Massarani, et al., 2012)
*Grewia erythraea Schweinf*.	Tiliaceae	Leaves	Methanol	*T.c* *T.b.b*	8.2 μg/ml 2.6 μg/ml	27.2 μg/ml	(Al‐Musayeib, Mothana, Al‐Massarani, et al., 2012)
*Iris albicans var. madonna Dykes*	Iridaceae	Leaves	Methanol	*T.c* *T.b.b*	>64.0 μg/ml 10.6 μg/ml	>64.0 μg/ml	(Al‐Musayeib, Mothana, Al‐Massarani, et al., 2012)
*Iris germanica L*.	Iridaceae	Roots	Methanol	*T.c* *T.b.b*	24.6 μg/ml 8.2 μg/ml	>64.0 μg/ml	(Al‐Musayeib, Mothana, Al‐Massarani, et al., 2012)
*Kanahia laniflora (Forssk.) R.Br*.	Iridaceae	Leaves	Methanol	*T.c* *T.b.b*	0.4 μg/ml 9.6 μg/ml	0.8 μg/ml	(Al‐Musayeib, Mothana, Al‐Massarani, et al., 2012)
*Kniphofia sumarae Deflers*	Asclepiadaceae	Leaves	Methanol	*T.c* *T.b.b*	31.4 μg/ml 5.9 μg/ml	7.4 μg/ml	(Al‐Musayeib, Mothana, Al‐Massarani, et al., 2012)
*Lavandula dentata var. candicans Batt*.	Liliaceae	Leaves	Methanol	*T.c* *T.b.b*	7.9 μg/ml 3.0 μg/ml	29.6 μg/ml	(Al‐Musayeib, Mothana, Al‐Massarani, et al., 2012)
*Leucas inflata Benth*.	Labiatae	Leaves	Methanol	*T.c* *T.b.b*	>64.0 μg/ml 8.4 μg/ml	29.5 μg/ml	(Al‐Musayeib, Mothana, Al‐Massarani, et al., 2012)
*Nigella sativa var. hispidula Boiss*.	Ranuncul‐aceae	Seeds	Methanol	*T.c* *T.b.b*	>64.0 μg/ml >64.0 μg/ml	>64.0 μg/ml	(Al‐Musayeib, Mothana, Al‐Massarani, et al., 2012)
*Periploca aphylla Decne*.	Asclepiad‐aceae	Leaves/stems	Methanol	*T.c* *T.b.b*	8.1 μg/ml 7.1 μg/ml	23.9 μg/ml	(Al‐Musayeib, Mothana, Al‐Massarani, et al., 2012)
*Phoenix dactylifera L*.	Arecaceae	Seeds	Methanol	*T.c* *T.b.b*	46.5 μg/ml 36.2 μg/ml	>64.0 μg/ml	(Al‐Musayeib, Mothana, Al‐Massarani, et al., 2012)
*Plectranthus barbatus var. grandis (L.H.Cramer) Lukhoba & A.J.Paton*	Labiatae	Leaves	Methanol	*T.c* *T.b.b*	23.3 μg/ml 2.6 μg/ml	32.9 μg/ml	(Al‐Musayeib, Mothana, Al‐Massarani, et al., 2012)
*Prosopis juliflora var. horrida (Kunth) Burkart*	Leguminosae	Fruits	Methanol	*T.c* *T.b.b*	10.4 μg/ml 2.0 μg/ml	49.8 μg/ml	(Al‐Musayeib, Mothana, Al‐Massarani, et al., 2012)
*Pulicaria inuloides (Poir.) DC*.	Labiatae	Leaves	Methanol	*T.c* *T.b.b*	31.7 μg/ml 7.8 μg/ml	>64.0 μg/ml	(Al‐Musayeib, Mothana, Al‐Massarani, et al., 2012)
*Punica granatum L*.	Punicaceae	Fruits	Methanol	*T.c* *T.b.b*	35.2 μg/ml 34.3 μg/ml	>64.0 μg/ml	(Al‐Musayeib, Mothana, Al‐Massarani, et al., 2012)
*Rhus retinorrhaea Steud. ex A.Rich*.	Anacardiaceae	Leaves	Methanol	*T.c* *T.b.b*	30.5 μg/ml 34.0 μg/ml	53.2 μg/ml	(Al‐Musayeib, Mothana, Al‐Massarani, et al., 2012)
*Ribes nigrum L*.	Grossulari‐aceae	Fruits	Methanol	*T.c* *T.b.b*	>64.0 μg/ml >64.0 μg/ml	>64.0 μg/ml	(Al‐Musayeib, Mothana, Al‐Massarani, et al., 2012)
*Salvadora persica var. persica*	Sallvador‐aceae	Leaves/stems	Methanol	*T.c* *T.b.b*	30.1 μg/ml 32.0 μg/ml	>64.0 μg/ml	(Al‐Musayeib, Mothana, Al‐Massarani, et al., 2012)
*Tagetes minuta L*.	Asteraceae	Leaves	Methanol	*T.c* *T.b.b*	9.2 μg/ml 2.2 μg/ml	>64.0 μg/ml	(Al‐Musayeib, Mothana, Al‐Massarani, et al., 2012)
*Tarconanthus camphoratus L*.	Asteraceae	Leaves	Methanol	*T.c* *T.b.b*	>64.0 μg/ml >64.0 μg/ml	>64.0 μg/ml	(Al‐Musayeib, Mothana, Al‐Massarani, et al., 2012)
*Teucrium yemense Deflers*	Labiatae	Leaves	Methanol	*T.c* *T.b.b*	30.5 μg/ml 7.1 μg/ml	27.2 μg/ml	(Al‐Musayeib, Mothana, Al‐Massarani, et al., 2012)
*Vernonia leopoldi (Sch.Bip. ex Walp.) Vatke*	Asteraceae	Leaves	Methanol	*T.c* *T.b.b*	9.2 μg/ml 8.0 μg/ml	30.1 μg/ml	(Al‐Musayeib, Mothana, Al‐Massarani, et al., 2012)
*Zingiber officinale var. cholmondeleyi F.M.Bailey*	Zingiber‐aceae	Roots	Methanol	*T.c* *T.b.b*	>64.0 μg/ml 39.4 μg/ml	34.3 μg/ml	(Al‐Musayeib, Mothana, Al‐Massarani, et al., 2012)

### 3.5 European plants

**FIGURE 4:**
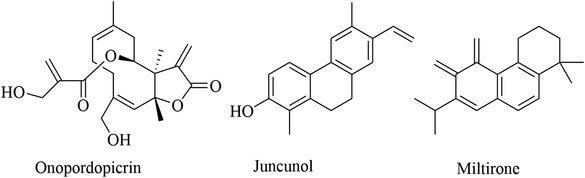
Chemical structures of isolated compounds from Europe medicinal plants

A total of 27 plants studied in Europe have been extracted from the literature. Of these, only three plants have bioactive compounds. The milestone was the most efficient bioactive compound with a minimum inhibitory concentration of 0.5 µg/ml (Ślusarczyk et al., 2011). *Trypanosoma brucei* was the most studied parasite, and a large variety of ethnobotanical families were included (Table 4 and Figure 4).

**TABLE 4:** Plants assessed for anti‐trypanosomal activity which the EC50 and the cytotoxicity values are known

Scientific name	Family	Part(s) used	Solvent	Bioactive compound	Model	Sub species	EC50	CC_50_	Country	References
*Arctium nemorosum Lej*.	Asteraceae	Leaf	Methanol	Onopordopicrin	In vitro	*T.b.r*	0.37 μM	3.06 μM	Switzerland	(Zimmermann et al., 2012)
*Arnica montana L*.	‎Asteraceae	Leaf	DCM	‐	In vitro	*T.b.r*	1.12 μg/ml	12.1 μg/ml	Germany	(Llurba‐Montesino et al., 2015)
*Callitris neocaledonica Dümmer*	Cupressaceae	Wood	Water	‐	In vitro	*T.b.b*	>50 μg/ml	>50 μg/ml	France	(Desrivot et al., 2007)
*Callitris sulcata (Parl.) Schltr*.	Cupressaceae	Wood	Water	‐	In vitro	*T.b.b*	>50 μg/ml	>50 μg/ml	France	(Desrivot et al., 2007)
*Citrus macroptera Montrouz*.	Rutaceae	Leaves	Water	‐	In vitro	*T.b.b*	>50 μg/ml	‐	France	(Desrivot et al., 2007)
*Crinum stuhlmannii subsp. delagoense (I.Verd.) Kwembeya & Nordal*	Amaryllidaceae	Leaves	Ethanol	‐	In vitro	*T.c*	0.70 μM	21.87 μM	Spain	(Martinez‐Peinado et al., 2020)
*Curcuma longa L*.	Zingiberaceae	Leaves	Water	‐	In vitro	*T.b.b*	>50 μg/ml	‐	France	(Desrivot et al., 2007)
*Dodonea viscosa L*.	Sapindaceae	Leaves	Ethanol	‐	In vitro	*T.b.b*	61.4 μg/ml	‐	France	(Desrivot et al., 2007)
*Eugenia uniflora L*	Myrtaceae	Bark	Water	‐	In vitro	*T.b.b*	>50 μg/ml	‐	France	(Desrivot et al., 2007)
*Eugenia uniflora L*.	Moraceae	Leaves	Methanol	‐	In vitro	*T.b.b*	46 μg/ml	‐	France	(Desrivot et al., 2007)
*Hernandia cordigera Vieill*.	Hernandiaceae	Bark	DCM	‐	In vitro	*T.b.b*	48 μg/ml	‐	France	(Desrivot et al., 2007)
*Homalium deplanchei Warb*.	Flacourtiaceae	Bark	DCM	‐	In vitro	*T.b.b*	>50 μg/ml	‐	France	(Desrivot et al., 2007)
*Hyacinthoides non‐scripta (L.) Chouard ex Rothm*.	Asparagaceae	Flowers	Methanol	‐	In vitro	*T.b.b*	11.1 μg/ml	‐	UK	(Raheem et al., 2019)
*Juncus acutus subsp. acutus*	Juncaceae	Leaves	DCM	Juncunol	In vitro	*T.c*	4.1 μg/ml	6.0 μg/ml	Portugal	(Oliveira et al., 2016)
*Manilkara dissecta (L.f.) Dubard*	Sapotaceae	Leaves	DCM	‐	In vitro	*T.b.b*	>50 μg/ml	‐	France	(Desrivot et al., 2007)
*Murraya crenulata (Turcz.) Oliv*.	Rutaceae	Bark	Hexane	‐	In vitro	*T.b.b*	27.6 μg/ml	‐	France	(Desrivot et al., 2007)
*Myoporum crassifolium G.Forst*.	Myoporaceae	Wood	Water	‐	In vitro	*T.b.b*	16 μg/ml	‐	France	(Desrivot et al., 2007)
*Myoporum tenuifolium G.Forst*.	Myoporaceae	Leaves	DCM	‐	In vitro	*T.b.b*	>50 μg/ml	‐	France	(Desrivot et al., 2007)
*Myristica fatua Houtt*.	Myristicacae	Almonds	DCM	‐	In vitro	*T.b.b*	0.5 μg/ml	‐	France	(Desrivot et al., 2007)
*Narcissus broussonetii var. grandiflorus Batt. & Trab*.	Amaryllidaceae	Leaves	Ethanol	‐	In vitro	*T.c*	0.495 μM	5.21 μM	Espain	(Martinez‐Peinado et al., 2020)
*Premna serratifolia L*.	Lamiaceae	Bark	DCM	‐	In vitro	*T.b.b*	>50 μg/ml	‐	France	(Desrivot et al., 2007)
*Prumnopytis ferruginoides L*.	Podocarpaceae	Leaves	Water	‐	In vitro	*T.b.b*	>50 μg/ml	‐	France	(Desrivot et al., 2007)
*Salvia officinalis subsp. gallica (W.Lippert) Reales, D.Rivera & Obón*	‎Lamiaceae	Leaves	DCM	‐	In vitro	*T.b.r*	1.86 μg/ml	32.3 μg/ml	Germany	(Llurba‐Montesino et al., 2015)
*Salvia miltiorrhiza var. charbonnelii (H.Lév.) C.Y.Wu*	Lamiaceae	Roots	DCM	Miltirone	In vitro	*T.b.r*	0.5 μg/ml	1.3 μg/ml	Switzerland	(Ślusarczyk et al., 2011)
*Scaevola balansae Guillaumin*	Goodneniaceae	Bark	DCM	‐	In vitro	*T.b.b*	39 μg/ml	‐	France	(Desrivot et al., 2007)
*Valeriana officinalis subsp. collina (Wallr.) Nyman*	Caprifoliaceae	Leaves	Ethanol	‐	In vitro	*T.c*	5.87 μg/ml	5.28 μg/ml	Germany	(Llurba‐Montesino et al., 2015)
*Wollastonia biflora (L.) DC*.	Asteraceae	Leaves	DCM	‐	In vitro	*T.b.b*	>100 μg/ml	‐	France	(Desrivot et al., 2007)

### 3.6 Latin America

**FIGURE 5:**
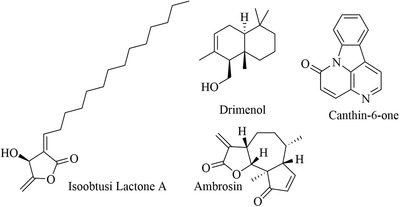
Chemical structures of isolated compounds from Latin America medicinal plants

A total of 165 plants have been reported throughout the literature, and just four have their isolated known compound. Researchers from South America have contributed to the investigation of anti‐trypanosomal plants. This result corroborates the scientometric analysis of global trypanosomiases research from 1988 to 2017, showing that South America ranked second behind Europe for contributions to trypanosomiases research (Hassan et al., 2020). The crude extracts of *Anthemis tinctoria* (semi‐purified), *Caseria sylvestris* (hexane) and *Ranunculus sceleratus* (ethanol) showed inhibitory activity against *Trypanosoma cruzi* with a minimum inhibitory concentration of 0.2, 0.3 and 0.7 µg/ml, respectively. For many plants, parasite growth inhibition is generally reported; thus, the minimum inhibitory concentration remains unknown (Table 5 and Figure 5).

**TABLE 5:** Plants assessed for anti‐trypanosomal activity which the EC50 values are known

Scientific name	Family	Part(s) used	Solvent	Bioactive compound	Model	Sub species	EC50	CC_50_	Country	References
*Abuta pahni (Mart.) Krukoff & Barneby*	Menisper maceae	Stems	petroleum ether, chloroform, ethyl acetate or 50% ethanol	‐	In vitro	*T.c*	100 μg/ml	‐	Bolivia	(Fournet et al., 1994)
*Acnistus arborescens (L.) Schltdl*.	Solanaceae	Leaf	Ethanol	‐	In vitro	*T.c*	4 μg/ml	‐	Panama	(Calderón et al., 2010)
*Aechmea distichantha var. glaziovii (Baker) L.B.Sm*.	Bromeliaceae	Leaf	Methanol	‐	In vitro	*T.c*	48 μg/ml	‐	Panama	(Calderón et al., 2010)
*Aiouea trinervis Meisn*.	Lauraceae	Leaf	Ethanol	Isoobtusi‐Lactone A	In vitro	*T.c*	2.75 μg/ml	156.45 μg/ml	Brazil	(Nunes et al., 2020)
*Angelica dahurica (Hoffm.) Benth. & Hook.f. ex Franch. & Sav*.	Apiaceae	Root	Ethanol	‐	In vitro	*T.c*	14.5 μg/ml	‐	Argentina	(Schinella et al., 2002)
*Angelica pubescens f. biserrata R.H.Shan & C.Q.Yuan*	Apiaceae	Root	Ethanol	‐	In vitro	*T.c*	14.9 μg/ml	‐	Argentina	(Schinella et al., 2002)
*Angelica sinensis (Oliv.) Diels*	Apiaceae	Wood	Ethanol	‐	In vitro	*T.c*	19.4 μg/ml	‐	Argentina	(Schinella et al., 2002)
*Annona crassiflora Mart*.	Annonaceae	Root bark	Ethanol	‐	In vitro	*T.c*	5.9 μg/ml	‐	Brazil	(Mesquita et al., 2005)
*Annona muricata L*.	Annonaceae	Leaf	Ethanol		In vitro	*T.c*	10 μg/ml	‐	Panama	(Calderón et al., 2010)
*Anomospermum chloranthum subsp. occidentale (Cuatrec.) Krukoff & Barneby*	Menisper maceae	Leaf	Alkaloid	‐	In vitro	*T.c*	100 μg/ml	‐	Bolivia	(Fournet et al., 1994)
*Anthemis tinctoria subsp. australis R.Fern*.	‎Asteraceae	Flowers	Semi‐purified	‐	In vitro	*T.c*	0.2 μg/ml	7.0 μg/ml	Brazil	(Bittencourt et al., 2011)
*Astragalus pehuenches Niederl*.	Fabaceae	Bark	Methanol	‐	In vitro	*T.c*	> 50 μg/ml	‐	Panama	(Calderón et al., 2010)
*Ardisia densiflora Krug & Urb*.	Myrsinaceae	Leaf	Ethanol	‐	In vitro	*T.c*	> 50 μg/ml	‐	Panama	(Calderón et al., 2010)
*Argemone subfusiformis Ownbey*	Papaveraceae	Fruit	Methanol	‐	In vitro	*T.c*	10 μg/ml	‐	Panama	(Calderón et al., 2010)
*Aristoloquia pilosa L*.	Aristolochiaceae	Stem	Hexane	‐	In vitro	*T.c*	100%	‐	Peru	(González‐Coloma et al., 2012)
*Artemisia mexicana Willd*.	Asteraceae	Aerial parts	Methanol	‐	In vitro	*T.c*	39.25 μg/ml	‐	Mexico	(Molina‐Garza et al., 2014)
*Atractyloides macrocephala L*.	Asteraceae	Root	Ethanol	‐	In vitro	*T.c*	23.0 μg/ml	‐	Argentina	(Schinella et al., 2002)
*Astragalus membranaceus (Fisch.) Bunge*	Fabaceae	Root	Water	‐	In vitro	*T.c*	13.5 μg/ml	‐	Argentina	(Schinella et al., 2002)
*Astronium fraxinifolium Schott*	Anacardiaceae	Stems bark	Hexane	‐	In vitro	*T.b.r*	16.4 μg/ml	>100 μg/ml	Brazil	(Charneau et al., 2016)
*Baccharis notosergila Griseb*.	Asteracea	Aerial parts	Methanol	‐	In vitro	*T.c*	>50 μg/ml	‐	Panama	(Calderón et al., 2010)
*Baccharis trinervis var. cinerea (DC.) Baker*	Asteraceae	Aerial parts	Ethanol	‐	In vitro	*T.c*	> 50 μg/ml	‐	Panama	(Calderón et al., 2010)
*Berberis conferta var. boliviana (Lechl.) C.K.Schneid*.	Berberidaceae	Stems	Alkaloid	‐	In vitro	*T.c*	75 μg/ml	‐	Bolivia	(Fournet et al., 1994)
*Berberis microphylla G.Forst*.	Berberidaceae	Aerial parts	Methanol	‐	In vitro	*T.c*	38.4 μg/ml	‐	Chile	(Muñoz et al., 2013)
*Blepharocalyx salicifolius (Kunth) O.Berg*	Myrtaceae	Leaves	Ethanol	‐	In vitro	*T.c*	37.3 μg/ml	55.1 μg/ml	Brazil	(Charneau et al., 2016)
*Bocconia integrifolia var. mexicana DC*.	Papaveraceae	Leaf	Ethanol	‐	In vitro	*T.c*	> 50 μg/ml	‐	Panama	(Calderón et al., 2010)
*Bourreria huanita (Lex.) Hemsl*.	Boraginaceae	Leaf	Ethanol	‐	In vitro	*T.c*	> 50 μg/ml	‐	Panama	(Calderón et al., 2010)
*Bourreria spathulata (Miers) Hemsl*.	Boraginaceae	Leaf	Methanol	‐	In vitro	*T.c*	30 μg/ml	‐	Panama	(Calderón et al., 2010)
*Brunfelsia grandiflora D.Don*	Solanaceae	Stem	Hexane	‐	In vitro	*T.c*	98%	‐	Peru	(González‐Coloma et al., 2012)
*Caesalpinia paraguariensis (Parodi) Burkart*	Fabaceae	Leaf	Ethanol	‐	In vitro	*T.c*	10 μg/ml	‐	Panama	(Calderón et al., 2010)
*Calea jamaicensis var. jamaicensis*	Asteraceae	Aerial parts	Ethanol	‐	In vitro	*T.c*	30 μg/ml	‐	Panama	(Calderón et al., 2010)
*Calea peruviana (Kunth) Benth. ex S.F.Blake*	Asteraceae	Leaf	Ethanol	‐	In vitro	*T.c*	> 50 μg/ml	‐	Panama	(Calderón et al., 2010)
*Capraria biflora f. hirta Loes*.	Scrophulariaceae	Aerial parts	Ethanol	‐	In vitro	*T.c*	46 μg/ml	‐	Panama	(Calderón et al., 2010)
*Capparis salicifolia Griseb*.	Capparaceae	Leaf	Ethanol	‐	In vitro	*T.c*	39 μg/ml	‐	Panama	(Calderón et al., 2010)
*Cardiopetalum calophyllum Schltdl*.	Annonaceae	Stem bark	Hexane	‐	In vitro	*T.c*	60.4 μg/ml	‐	Brazil	(Mesquita et al., 2005)
*Cardiopetalum calophyllum Schltdl*.	Annonaceae	Leaves	Alkaloidal	‐	In vitro	*T.c*	100 μg/ml	‐	Bolivia	(Fournet et al., 1994)
*Casearia sylvestris var. lingua (Cambess.) Eichler*	Flacourtiaceae	Root bark	Hexane	‐	In vitro	*T.c*	0.3 μg/ml	‐	Brazil	(Mesquita et al., 2005)
*Cedrela odorata var. xerogeiton Rizzini & Heringer*	Meliaceae	Bark	Hexane	‐	In vitro	*T.c*	100%	‐	Peru	(González‐Coloma et al., 2012)
*Cestrum parqui (Lam.) L'Hér*.	Solanaceae	Aerial parts	Ethanol	‐	In vitro	*T.c*	> 50 μg/ml	‐	Panama	(Calderón et al., 2010)
*Chamaecrista desvauxii (Collad.) Killip*	Caesalpiniaceae	Leaves	Ethanol	‐	In vitro	*T.c*	>80%	‐	Brazil	(Charneau et al., 2016)
*Chondodendron tomentosum L*.	Menispermaceae	Bark	Chloroform	‐	In vitro	*T.c*	100%	‐	Peru	(González‐Coloma et al., 2012)
*Chromolaena leivensis (Hieron.) R.M.King & H.Rob*.	Asteraceae	Aerial parts	Ethanol	‐	In vitro	*T.c*	8 μg/ml	‐	Panama	(Calderón et al., 2010)
*Cinchona pubescens var. heterophylla Pav. ex DC*.	Rubiaceae	Leaf	Methanol	‐	In vitro	*T.c*	> 50 μg/ml	‐	Panama	(Calderón et al., 2010)
*Cissampelos tropaeolifolia var. fluminensis (Eichler) Diels*	Menispermaceae	Leaf	Ethanol	‐	In vitro	*T.c*	> 50 μg/ml	‐	Panama	(Calderón et al., 2010)
*Clarisia biflora Ruiz & Pav*.	Moraceae	Aerial parts	Ethanol	‐	In vitro	*T.c*	25 μg/ml	‐	Panama	(Calderón et al., 2010)
*Clematis campestris var. mendocina (Phil.) Hauman & Irigoyen*	Ranunculaceae	Flowers	Methanol	‐	In vitro	*T.c*	> 50 μg/ml	‐	Panama	(Calderón et al., 2010)
*Combretum laxum var. epiphyticum (Pittier) Croat*	Combretaceae	Aerial parts	Methanol	‐	In vitro	*T.c*	34 μg/ml	‐	Panama	(Calderón et al., 2010)
*Codonopsis pilosula var. glaberrima (Nannf.) P.C.Tsoong*	Campanulaceae	Roots	Water	‐	In vitro	*T.c*	20.8 μg/ml	‐	Argentina	(Schinella et al., 2002)
*Connarus suberosus var. fulvus (Planch.) Forero*	Connaraceae	Roots woods	Hexane	‐	In vitro	*T.b.r*	1.7 μg/ml	2.6 μg/ml	Brazil	(Charneau et al., 2016)
*Cordia cylindrostachya (Ruiz & Pav.) Roem. & Schult*.	Boraginaceae	Leaf	Ethanol	‐	In vitro	*T.c*	35 μg/ml	‐	Panama	(Calderón et al., 2010)
*Crataegus pubescens (C.Presl) C.Presl*	Rosaceae	Fruit	Ethanol	‐	In vitro	*T.c*	> 50 μg/ml	‐	Panama	(Calderón et al., 2010)
*Critonia morifolia (Mill.) R.M.King & H.Rob*.	Asteraceae	Fruit	Ethanol	‐	In vitro	*T.c*	29 μg/ml	‐	Panama	(Calderón et al., 2010)
*Curcuma aromatic L*.	Zingiberaceae	Rhizome	Water	‐	In vitro	*T.c*	21.4 μg/ml	‐	Argentina	(Schinella et al., 2002)
*Cymbopogon citratus (DC.) Stapf*	Poaceae	Aerial parts	Methanol	‐	In vitro	*T.c*	68.25 μg/ml	‐	Mexico	(Molina‐Garza et al., 2014)
*Dalbergia ecastaphyllum (L.) Taub*.	Fabaceae	Plant resin	Hydroethanol	‐	In vitro	*T.c*	88.86 μg/ml	228.02 μg/ml	Brazil	(Regueira‐Neto et al., 2018)
*Drimys winteri J.R.Forst. & G.Forst*.	Winteraceae	Aerial parts	DCM	Drimenol	In vitro	*T.c*	25.1 μg/ml	‐	Chile	(Muñoz et al., 2013)
*Duguetia furfuracea (A.St.‐Hil.) Saff*.	Annonaceae	Root bark	Hexane	‐	In vitro	*T.c*	6.6 μg/ml	‐	Brazil	(Mesquita et al., 2005)
*Egletes viscosa var. dissecta Shinners*	Asteraceae	Whole plants	Ethanol	‐	In vitro	*T.c*	38 μg/ml	‐	Panama	(Calderón et al., 2010)
*Eirmocephala brachiata H.Rob*.	Asteraceae	Leaf	Ethanol	‐	In vitro	*T.c*	33 μg/ml	‐	Panama	(Calderón et al., 2010)
*Eryngium heterophyllum Engelm*.	Apiaceae	Aerial parts	Methanol	‐	In vitro	*T.c*	11.24 μg/ml	‐	Mexico	(Molina‐Garza et al., 2014)
*Euterpe precatoria var. longivaginata (Mart.) A.J.Hend*.	Arecaceae	Root	Methanol	‐	In vitro	*T.c*	> 50 μg/ml	‐	Panama	(Calderón et al., 2010)
*Forsythia suspensa (Thunb.) Vahl*	Oleaceae	Fruit	Methanol	‐	In vitro	*T.c*	19.1 μg/ml	‐	Argentina	(Schinella et al., 2002)
*Fuchsia boliviana var. luxurians I.M.Johnst*.	Onagraceae	Leaf	Ethanol	‐	In vitro	*T.c*	> 50 μg/ml	‐	Panama	(Calderón et al., 2010)
*Galium latoramosum Clos*	Rubiaceae	Aerial parts	Methanol	‐	In vitro	*T.c*	> 50 μg/ml	‐	Panama	(Calderón et al., 2010)
*Gnaphalium gaudichaudianum var. gaudichaudianum*	Asteraceae	Aerial parts	Methanol	‐	In vitro	*T.c*	36 μg/ml	‐	Panama	(Calderón et al., 2010)
*Gochnatia glutinosa (D.Don) D.Don ex Hook. & Arn*.	Asteraceae	Aerial parts	Methanol	‐	In vitro	*T.c*	20 μg/ml	‐	Panama	(Calderón et al., 2010)
*Haematoxylum brasiletto H.Karst*.	Fabaceae	Bark	Methanol	‐	In vitro	*T.c*	7.92 μg/ml	‐	Mexico	(Molina‐Garza et al., 2014)
*Haplophyllum hispanicum Spach*	Rutaceae	Fruit	Ethanol	‐	In vitro	*T.c*	8.5 μg/ml	16.7	Argentina	(Schinella et al., 2002)
*Hauya lucida Donn.Sm. & Rose*	Onagraceae	Aerial parts	Methanol	‐	In vitro	*T.c*	32 μg/ml	‐	Panama	(Calderón et al., 2010)
*Helichrysum italicum (Roth) G.Don*	Rutaceae	Aerial parts	Methanol	‐	In vitro	*T.c*	23.0 μg/ml	‐	Argentina	(Schinella et al., 2002)
*Himatanthus obovatus (Müll.Arg.) Woodson*	Apocynaceae	Root wood	Ethanol	‐	In vitro	*T.c*	15.7 μg/ml	‐	Brazil	(Mesquita et al., 2005)
*Ilex guayusa Loes*.	Aquifoliaceae	Leaf	Ethanol	‐	In vitro	*T.c*	47 μg/ml	‐	Panama	(Calderón et al., 2010)
*Inula viscosa (L.) Aiton*	Asteraceae	Aerial parts	Ethanol	‐	In vitro	*T.c*	27.5 μg/ml	‐	Argentina	(Schinella et al., 2002)
*Ipomoea carnea subsp. carnea*	Convolvulaceae	Leaf	Ethanol	‐	In vitro	*T.c*	48 μg/ml	‐	Panama	(Calderón et al., 2010)
*Jacaranda mimosifolia D.Don*	Bignoniaceae	Leaf	Methanol	‐	In vitro	*T.c*	> 50 μg/ml	‐	Panama	(Calderón et al., 2010)
*Kageneckia oblonga Ruiz & Pav*.	Rosaceae	Aerial parts	Methanol	‐	In vitro	*T.c*	35.7 μg/ml	‐	Chile	(Muñoz et al., 2013)
*Larrea cuneifolia Cav*.	Zygophyllaceae	Aerial parts	Methanol	‐	In vitro	*T.c*	40 μg/ml	‐	Panama	(Calderón et al., 2010)
*Lippia graveolens Kunth*	Verbenaceae	Leaf	Ethanol	‐	In vitro	*T.c*	13 μg/ml	‐	Panama	(Calderón et al., 2010)
*Lithrea caustica Hook. & Arn*.	Anacardiaceae	Aerial parts	Methanol	‐	In vitro	*T.c*	> 50 μg/ml	‐	Panama	(Calderón et al., 2010)
*Lentinus edodes L*.	Marasmiaceae	Sclerotium	Water	‐	In vitro	*T.c*	26.8 μg/ml	‐	Argentina	(Schinella et al., 2002)
*Lozania pittieri (S.F.Blake) L.B.Sm*.	Flacourtiaceae	Leaf	Methanol	‐	In vitro	*T.c*	30 μg/ml	‐	Panama	(Calderón et al., 2010)
*Lycium cuneatum Dammer*	Solanaceae	Aerial parts	Ethanol	‐	In vitro	*T.c*	29 μg/ml	‐	Panama	(Calderón et al., 2010)
*Maianthemum paludicola LaFrankie*	Convallariaceae	Whole plants	Methanol	‐	In vitro	*T.c*	5 μg/ml	‐	Panama	(Calderón et al., 2010)
*Mandevilla antennacea (A.DC.) K.Schum*.	Apocynaceae	Leaves stems	Ethanol	‐	In vitro	*T.c*	100 μg/ml	‐	Bolivia	(Fournet et al., 1994)
*Marrubium vulgare subsp. apulum (Ten.) H.Lindb*.	Lamiaceae	Aerial parts	Methanol	‐	In vitro	*T.c*	22.66 μg/ml	‐	Mexico	(Molina‐Garza et al., 2014)
*Matayba guianensis Aubl*.	Sapindaceae	Stems bark	Hexane	‐	In vitro	*T.c*	17.8 μg/ml	‐	Brazil	(Mesquita et al., 2005)
*Miconia buxifolia Naudin*	Melastomataceae	Leaf	Ethanol	‐	In vitro	*T.c*	> 50 μg/ml	‐	Panama	(Calderón et al., 2010)
*Mikania periplocifolia Hook. & Arn*.	Asteraceae	Aerial parts	Methanol	‐	In vitro	*T.c*	> 50 μg/ml	‐	Panama	(Calderón et al., 2010)
*Munnozia maronii (André) H.Rob*.	Asteraceae	Leaves	Ethanol	‐	In vitro	*T.c*	25 μg/ml	‐	Bolivia	(Fournet et al., 1994)
*Myrsine guianensis (Aubl.) Kuntze*	Myrsinaceae	Leaves	Hexane	‐	In vitro	*T.c*	65.0 μg/ml	107.1 μg/ml	Brazil	(Charneau et al., 2016)
*Myrcianthes rhopaloides (Kunth) McVaugh*	Myrtaceae	Leaves	Methanol	‐	In vitro	*T.c*	24 μg/ml	‐	Panama	(Calderón et al., 2010)
*Nicotiana glauca var. angustifolia Comes*	Solanaceae	Aerial parts	Methanol	‐	In vitro	*T.c*	38 μg/ml	‐	Panama	(Calderón et al., 2010)
*Paeonia lactiflora var. lactiflora*	Paeoniaceae	Root	Water	‐	In vitro	*T.c*	27.9 μg/ml	‐	Argentina	(Schinella et al., 2002)
*Parthenium hysterophorus L*.	Asteraceae	Leaves	DCM	Ambrosin	In vitro	*T.b.b*	67.1 μg/ml	11.46 μg/ml	Mexico	(Sepúlveda‐Robles et al., 2019)
*Parietaria debilis var. ceratosantha Wedd*.	Urticaceae	Aerial parts	Methanol	‐	In vitro	*T.c*	> 50 μg/ml	‐	Panama	(Calderón et al., 2010)
*Paullinia clavigera Schltdl*.	Sapindaceae	Bark	Chloroform	‐	In vitro	*T.c*	100%	‐	Peru	(González‐Coloma et al., 2012)
*Persea americana var. americana*	Lauraceae	Leaf	Methanol	‐	In vitro	*T.c*	65.51 μg/ml	‐	Mexico	(Molina‐Garza et al., 2014)
*Phellodendron amurense var. sachalinense F. Schmidt*	Rutaceae	Root bark	Methanol	‐	In vitro	*T.c*	11.3 μg/ml	‐	Argentina	(Schinella et al., 2002)
*Phyla betulifolia (Kunth) Greene*	Verbenaceae	Whole plant	Methanol	‐	In vitro	*T.c*	30 μg/ml	‐	Panama	(Calderón et al., 2010)
*Phytolacca bogotensis Kunth*	Phytolaccaceae	Aerial parts	Methanol	‐	In vitro	*T.c*	> 50 μg/ml	‐	Panama	(Calderón et al., 2010)
*Phytolacca tetramera Hauman*	Phytolaccaceae	Aerial parts	Methanol	‐	In vitro	*T.c*	> 50 μg/ml	‐	Panama	(Calderón et al., 2010)
*Piper acutifolium Ruiz & Pav*.	Piperaceae	Leaf	DCM	‐	In vitro	*T.c*	39 μg/ml	‐	Panama	(Calderón et al., 2010)
*Piper aduncum var. brachyarthrum (Trel.) Yunck*.	Piperaceae	Leaf	DCM	‐	In vitro	*T.c*	38 μg/ml	‐	Panama	(Calderón et al., 2010)
*Piper aeruginosibaccum Trel*.	Piperaceae	Leaf	Ethanol	‐	In vitro	*T.c*	12 μg/ml	‐	Panama	(Calderón et al., 2010)
*Piper barbatum var. andicolum (Kunth) Trel. & Yunck*.	Piperaceae	Leaf	Ethanol	‐	In vitro	*T.c*	10 μg/ml	‐	Panama	(Calderón et al., 2010)
*Piper dilatatum f. dilatatifolium (Trel. & Yunck.) Steyerm*.	Piperaceae	Leaf	DCM	‐	In vitro	*T.c*	31 μg/ml	‐	Panama	(Calderón et al., 2010)
*Piper elongatum var. brachyarthrum Trel*.	Piperaceae	Leaf	DCM	‐	In vitro	*T.c*	36 μg/ml	‐	Panama	(Calderón et al., 2010)
*Piper glabratum Kunth*	Piperaceae	Leaf	DCM	‐	In vitro	*T.c*	25 μg/ml	‐	Panama	(Calderón et al., 2010)
*Piper hispidum var. gamboanum C. DC*.	Piperaceae	Leaf	DCM	‐	In vitro	*T.c*	26 μg/ml	‐	Panama	(Calderón et al., 2010)
*Piper holtonii var. parvispicum Yunck*.	Piperaceae	Root	Ethanol	‐	In vitro	*T.c*	10 μg/ml	‐	Panama	(Calderón et al., 2010)
*Piper longestylosum C. DC*.	Piperaceae	Leaf	DCM	‐	In vitro	*T.c*	> 50 μg/ml	‐	Panama	(Calderón et al., 2010)
*Piper abalienatum Trel*.	Piperaceae	Leaf	DCM	‐	In vitro	*T.c*	35 μg/ml	‐	Panama	(Calderón et al., 2010)
*Piper rusbyi C. DC*.	Piperaceae	Leaf	DCM	‐	In vitro	*T.c*	32 μg/ml	‐	Panama	(Calderón et al., 2010)
*Piper scabrum Willd. ex Kunth*	Piperaceae	Leaf	Ethanol	‐	In vitro	*T.c*	32 μg/ml	‐	Panama	(Calderón et al., 2010)
*Piper umbellatum var. glabrum C. DC*.	Piperaceae	Leaf	Ethanol	‐	In vitro	*T.c*	25 μg/ml	‐	Panama	(Calderón et al., 2010)
*Podanthus ovatifolius Lag*.	Asteraceae	Aerial parts	Methanol	‐	In vitro	*T.c*	40.1 μg/ml	‐	Chile	(Muñoz et al., 2013)
*Polygonum acuminatum Kunth*	Polygonaceae	Leaf	Methanol	‐	In vitro	*T.c*	> 50 μg/ml	‐	Panama	(Calderón et al., 2010)
*Polygonum ferrugineum var. patagonicum (Speg.) Macloskie*	Polygonaceae	Aerial parts	Methanol	‐	In vitro	*T.c*	37 μg/ml	‐	Panama	(Calderón et al., 2010)
*Poria cocos L*.	Polyporaceae	Sclerotium	Ethanol	‐	In vitro	*T.c*	16.8 μg/ml	‐	Argentina	(Schinella et al., 2002)
*Pouteria gardneri (Mart. & Eichler ex Miq.) Baehni*	Sapindaceae	Roots woods	Hexane	‐	In vitro	*T.c*	45.5 μg/ml	‐	Brazil	(Mesquita et al., 2005)
*Piscidia carthagenensis Jacq*.	Fabaceae	Aerial parts	Methanol	‐	In vitro	*T.c*	28 μg/ml	‐	Panama	(Calderón et al., 2010)
*Psidium laruotteanum Cambess*	‎Myrtaceae	Leaves	Hexane	‐	In vitro	*T.b.r*	3.9 μg/ml	>100 μg/ml	Brazil	(Charneau et al., 2016)
*Psittacanthus cordatus (Hoffmanns. ex Schult. f.) Blume*	Loranthaceae	Leaf	Ethanol	‐	In vitro	*T.c*	40 μg/ml	‐	Panama	(Calderón et al., 2010)
*Ranunculus sceleratus subsp. multifidus (Nutt.) Hultén*	Renonculaceae	Aerial parts	Ethanol	‐	In vitro	*T.c*	0.7 μg/ml	18.7 μg/ml	Argentina	(Schinella et al., 2002)
*Rauvolfia tetraphylla L*.	Apocynaceae	Root	Ethanol	‐	In vitro	*T.c*	> 50 μg/ml	‐	Panama	(Calderón et al., 2010)
*Rehmania glutinosa L*.	Oronbanchaceae	Root	Ethanol	‐	In vitro	*T.c*	24.5 μg/ml	‐	Argentina	(Schinella et al., 2002)
*Ruta chalepensis L*.	Rutaceae	Leaf	Methanol	‐	In vitro	*T.c*	72.30 μg/ml	‐	Mexico	(Molina‐Garza et al., 2014)
*Salvertia convallariodora A. St.‐Hil*.	Vochysiaceae	Leaves	Hexane	‐	In vitro	*T.b.g*	35.4 μg/ml	>100 μg/ml	Brazil	(Charneau et al., 2016)
*Schinus molle var. areira (L.) DC*.	Anarcadiaceae	Leaves	Methanol	‐	In vitro	*T.c*	16.31 μg/ml		Mexico	(Molina‐Garza et al., 2014)
*Scoparia dulcis L*.	Scrophulariaceae	Whole plants	Ethanol	‐	In vitro	*T.c*	4 μg/ml	‐	Panama	(Calderón et al., 2010)
*Scrophularia auriculata L*.	Scrofulariaceaes	Aerial parts	Ethanol	‐	In vitro	*T.c*	23.3 μg/ml	‐	Argentina	(Schinella et al., 2002)
*Scutellaria baicalensis f. albiflora H.W.Jen & Y.J.Chang*	Lamiaceae	Root	Methanol	‐	In vitro	*T.c*	7.5 μg/ml	28.7 μg/ml	Argentina	(Schinella et al., 2002)
*Sebastiania brasiliensis var. anisophylla Müll.Arg*.	Euphorbiaceae	Aerial parts	Methanol	‐	In vitro	*T.c*	> 50 μg/ml	‐	Panama	(Calderón et al., 2010)
*Sebastiania commersoniana (Baill.) L.B.Sm. & Downs*	Euphorbiaceae	Aerial parts	Methanol	‐	In vitro	*T.c*	> 50 μg/ml	‐	Panama	(Calderón et al., 2010)
*Solidago chilensis var. chilensis*	Asteraceae	Leaves	Methanol	‐	In vitro	*T.c*	32 μg/ml	‐	Panama	(Calderón et al., 2010)
*Sapranthus viridiflorus G.E. Schatz*	Annonaceae	Aerial parts	Methanol	‐	In vitro	*T.c*	25 μg/ml	‐	Panama	(Calderón et al., 2010)
*Sarcostemma gracile Decne*.	Asclepiadaceae	Aerial parts	Ethanol	‐	In vitro	*T.c*	42 μg/ml	‐	Panama	(Calderón et al., 2010)
*Schinus molle var. areira (L.) DC*.	Anacardiaceae	Aerial parts	Methanol	‐	In vitro	*T.c*	> 50 μg/ml	‐	Panama	(Calderón et al., 2010)
*Solanum actaeibotrys Rusby*	Solanaceae	Leaves	Ethanol	‐	In vitro	*T.c*	100 μg/ml	‐	Bolivia	(Fournet et al., 1994)
*Solanum cornifolium Dunal*	Solanaceae	Leaf	Ethanol	‐	In vitro	*T.c*	> 50 μg/ml	‐	Panama	(Calderón et al., 2010)
*Srevia yaconensis L*.	Asteraceae	Woods	Ethanol	‐	In vitro	*T.c*	50 μg/ml	‐	Bolivia	(Fournet et al., 1994)
*Styrax conterminus Donn.Sm*.	Styracaceae	Bark	Ethanol	‐	In vitro	*T.c*	> 50 μg/ml	‐	Panama	(Calderón et al., 2010)
*Tabebuia serratifolia (Vahl) G.Nicholson*	Bignoniaceae	Bark	Hexane	‐	In vitro	*T.c*	100 μg/ml	‐	Peru	(González‐Coloma et al., 2012)
*Tagetes caracasana Humb. ex Willd*.	Asteraceae	Leaves	Oil	‐	In vitro	*T.c*	4.56 μg/ml	25.73 μg/ml	Brazil	(Escobar et al., 2009)
*Tagetes filifolia subsp. filifolia*	Asteraceae	Aerial parts	Methanol	‐	In vitro	*T.c*	> 50 μg/ml	‐	Panama	(Calderón et al., 2010)
*Tagetes heterocarpha Rydb*.	Asteraceae	Leaves	Oil	‐	In vitro	*T.c*	12.84 μg/ml	43.03 μg/ml	Brazil	(Escobar et al., 2009)
*Tagetes lucida f. florida (Sweet) Voss*	Asteraceae	Leaves	Oil	‐	In vitro	*T.c*	18.94 μg/ml	>300 μg/ml	Brazil	(Escobar et al., 2009)
*Tagetes zypaquirensis Bonpl*.	Asteraceae	Leaves	Oil	‐	In vitro	*T.c*	21.30 μg/ml	126.40 μg/ml	Brazil	(Escobar et al., 2009)
*Terminalia triflora (Griseb.) Lillo*	Combretaceae	Aerial parts	Methanol	‐	In vitro	*T.c*	> 50 μg/ml	‐	Panama	(Calderón et al., 2010)
*Tradescantia zebrina var. flocculosa (G.Brückn.) D.R.Hunt*	Commelinaceae	Aerial parts	Hexane	‐	In vitro	*T.c*	96%	‐	Peru	(González‐Coloma et al., 2012)
*Tynanthus guatemalensis Donn.Sm*	Bignoniaceae	Stems	Ethanol	‐	In vitro	*T.c*	> 50 μg/ml	‐	Panama	(Calderón et al., 2010)
*Vatairea macrocarpa var. cinerascens (Benth.) Ducke*	Fabaceae	Roots woods	Hexane	‐	In vitro	*T.c*	32.6 μg/ml	>100 μg/ml	Brazil	(Charneau et al., 2016)
*Vernonia squamulosa Hook. & Arn*.	Asteraceae	Leaves	Petroleum	‐	In vitro	*T.c*	100 μg/ml	‐	Bolivia	(Fournet et al., 1994)
*Xylopia aromatica (Lam.) Mart*.	Annonaceae	Root woods	Hexane	‐	In vitro	*T.c*	21.6 μg/ml	‐	Brazil	(Mesquita et al., 2005)
*Zamia ulei subsp. lecointei (Ducke) Ducke*	Zamiaceae	Underground tuberous stem	Chloroform	‐	In vitro	*T.c*	92.5%	‐	Peru	(González‐Coloma et al., 2012)
*Zanthoxylum chiloperone var. angustifolium Engl*.	Rutaceae	Aerial parts	Alkaloidal	canthin‐6‐one	In vivo	*T.c* in Balb/c mice, 5 mg/kg/day	80–100% inhibition	‐	Paraguay	(Ferreira et al., 2007)
*Ziziphus mistol Griseb*.	Rhamnaceae	Leaf	Ethanol	‐	In vitro	*T.c*	25 μg/ml	‐	Panama	(Calderón et al., 2010)
*Zuccagnia punctata Cav*.	Fabaceae	Leaf	Ethanol	‐	In vitro	*T.c*	20 μg/ml	‐	Panama	(Calderón et al., 2010)

### 3.7 North America

A total of 29 plants have been identified in the literature. Interestingly, the lack of testing against *T. cruzi*, which is prevalent in the southern part of North America, was observed in this study. The plants showed excellent anti‐trypanosomal activity with a minimum inhibitory concentration of fewer than 10 µg/ml. The crude extracts of *Nuphar luteum* (0.42 µg/ml), *Hoita macrostachya* (0.48 µg/ml) and *Rhus integrifolia* (0.50 µg/ml) showed the highest activity against *Trypanosoma brucei* (Table 6).

**TABLE 6:** Plants assessed for anti‐trypanosomal application for which the EC50 values are known

Scientific name	Family	Part(s) used	Solvent	Bioactive compound	Model	Sub species	EC50	CC_50_	Country	References
*Acer rubrum subsp. carolinianum (Walter) W.Stone*	Sapindaceae	Leaf	Ethanol	‐	In vitro	T.b.b	2.88 μg/ml	‐	USA	(Jain et al., 2016)
*Alnus rubra f. pinnatisecta (Starker) Rehder*	Betulaceae	Bark	Ethanol	‐	In vitro	*T.b.b*	0.94 μg/ml	‐	USA	(Jain et al., 2016)
*Anogeissus leiocarpus (DC.) Guill. & Perr*.	Combretaceae	Root bark	Methanol	‐	In vitro	*T.b.b*	0.82 μg/ml	‐	USA	(Kenguele, 2009)
*Arctostaphylos viscida subsp. mariposa (Dudley) P.V.Wells*	Ericaceae	Leaf	Ethanol	‐	In vitro	*T.b.b*	2.88 μg/ml	‐	USA	(Jain et al., 2016)
*Boykinia major var. intermedia (A.Heller) Piper*	Saxifragaceae	Root	Ethanol	‐	In vitro	*T.b.b*	2.82 μg/ml	‐	USA	(Jain et al., 2016)
*Chrysolepis chrysophylla (Douglas ex Hook.) Hjelmq. *	Fagaceae	Flowers	Ethanol	‐	In vitro	*T.b.b*	2.89 μg/ml	‐	USA	(Jain et al., 2016)
*Coccoloba pubescens L*.	Polygonaceae	Stems	Ethanol	‐	In vitro	*T.b.b*	0.83 μg/ml	‐	USA	(Jain et al., 2016)
*Eriogonum fasciculatum var. polifolium (Benth.) Torr. & A.Gray*	Polygonaceae	Leaf	Ethanol	‐	In vitro	*T.b.b*	2.68 μg/ml	‐	USA	(Jain et al., 2016)
*Eriogonum umbellatum subsp. dumosum (Greene) S.Stokes*	Polygonaceae	Leaf stems	Ethanol	‐	In vitro	*T.b.b*	2.79 μg/ml	‐	USA	(Jain et al., 2016)
*Eucalyptus citriodora Hook*.	Myrtaceae	Leaf	Ethanol	‐	In vitro	*T.b.b*	2.91 μg/ml	‐	USA	(Jain et al., 2016)
*Fagara zanthoxyloides Lam*.	Rutaceae	Bark	Methanol	‐	In vitro	*T.b.b*	6.42 μg/ml	‐	USA	(Kenguele, 2009)
*Hamamelis virginiana f. parvifolia (Nutt.) Fernald*	Hamamelidaceae	Stem	Ethanol	‐	In vitro	*T.b.b*	2.54 μg/ml	‐	USA	(Jain et al., 2016)
*Hoita macrostachya (DC.) Rydb*.	Fabaceae	Leaf	Ethanol	‐	In vitro	*T.b.b*	0.48 μg/ml	‐	USA	(Jain et al., 2016)
*Juniperus communis subsp. alpina (*Schoop*, Büechi et al.) Celak*.	Cupressaceae	Leaf stem	Ethanol	‐	In vitro	*T.b.b*	2.40 μg/ml	‐	USA	(Jain et al., 2016)
*Leea rubra Blume ex Spreng*.	Vitaceae	Stem	Ethanol	‐	In vitro	*T.b.b*	1.62 μg/ml	‐	USA	(Jain et al., 2016)
*Lepechinia calycina var. glabella (A.Gray) Epling ex Munz*	Lamiaceae	Leaf	Ethanol	‐	In vitro	*T.b.b*	2.50 μg/ml	‐	USA	(Jain et al., 2016)
*Ligustrum sinense var. myrianthum (Diels) Hoefker*	Oleaceae	Leaf fruit	Ethanol	‐	In vitro	*T.b.b*	2.77 μg/ml	‐	USA	(Jain et al., 2016)
*Lyonia fruticosa (Michx.) G.S. Torr*.	Ericaceae	Stems	Ethanol	‐	In vitro	*T.b.b*	2.54 μg/ml	‐	USA	(Jain et al., 2016)
*Medinilla magnifica Lindl*.	Melastomataceae	Flowers fruit	Ethanol	‐	In vitro	*T.b.b*	2.25 μg/ml	‐	USA	(Jain et al., 2016)
*Nuphar lutea subsp. advena Kartesz & Gandhi*	Nymphaeaceae	Fruit	Ethanol	‐	In vitro	*T.b.b*	0.42 μg/ml	‐	USA	(Jain et al., 2016)
*Quercus alba f. latiloba (Sarg.) E.J.Palmer & Steyerm*.	Fagaceae	Bark	Ethanol	‐	In vitro	*T.b.b*	1.92 μg/ml	‐	USA	(Jain et al., 2016)
*Pseudocedrela kotschyi (Schweinf.) Harms*	Meliaceae	Root	Methanol	‐	In vitro	*T.b.b*	8.94 μg/ml	‐	USA	(Kenguele, 2009)
*Rhododendron occidentale (Torr. & A. Gray) A. Gray*	Ericaceae	Leaf	Ethanol	‐	In vitro	*T.b.b*	2.87 μg/ml	‐	USA	(Jain et al., 2016)
*Rhus integrifolia (Nutt.) Benth. & Hook. f. ex Rothr*.	Anacardiaceae	Leaf	Ethanol	‐	In vitro	*T.b.b*	0.50 μg/ml	‐	USA	(Jain et al., 2016)
*Ribes montigenum McClatchie*	Grossulariaceae	Stems	Ethanol	‐	In vitro	*T.b.b*	1.94 μg/ml	‐	USA	(Jain et al., 2016)
*Ribes speciosum Pursh*	Grossulariaceae	Leaf stems flowers	Ethanol	‐	In vitro	*T.b.b*	2.95 μg/ml	‐	USA	(Jain et al., 2016)
*Sabal minor (Jacq.) Pers*.	Arecaceae	Flowers	Ethanol	‐	In vitro	*T.b.b*	1.06 μg/ml	‐	USA	(Jain et al., 2016)
*Salvia spathacea Greene*	Lamiaceae	Stems	Ethanol	‐	In vitro	*T.b.b*	1.13 μg/ml	‐	USA	(Jain et al., 2016)
*Terminalia glaucescens Planch. ex Benth*.	Combretaceae	Root	Methanol	‐	In vitro	*T.b.b*	9.04 μg/ml	‐	USA	(Kenguele, 2009)

### 3.8 Oceania plants

Few studies have been found in the literature about the medicinal plants from Oceania with anti‐trypanosomal activity. This corroborates with the scientometric analysis of global trypanosomiases research from 1988 to 2017 which shows that Oceania researchers have contributed less than the others to trypanosomiases research in this region (Hassan et al., 2020). Only seven plants have been identified in the literature, of which just *Corydalis crispa* (4.63 µg/ml) showed activity against *Trypanosoma brucei* (Table 7).

**TABLE 7:** Plants assessed for anti‐trypanosomal activity

Scientific Name	Family	Part (s) used	Solvent	Bioactive compound	Model	Sub species	EC50	CC_50_	Country	References
*Aconitum laciniatum (Brühl) Stapf*	Ranunculaceae	Leaf	Methanol	‐	In vitro	*T.b.b*	>25 μg/ml	>25 μg/ml	Australia	(Wangchuk, 2014)
*Ajania nubigena (Wall.) C.Shih*	Compositae	Leaf	Methanol	‐	In vitro	*T.b.b*	>10 μg/ml	>10 μg/ml	Australia	(Wangchuk, 2014)
*Codonopsis bhutanica Ludlow*	Campanulaceae	Leaf	Methanol	‐	In vitro	*T.b.b*	>5 μg/ml	>5 μg/ml	Australia	(Wangchuk, 2014)
*Corydalis crispa var. laeviangula C.Y.Wu & H.Chuang*	Fumariaceae	Leaf	Methanol	‐	In vitro	*T.b.b*	4.63 μg/ml	12.5 μg/ml	Australia	(Wangchuk, 2014)
*Corydalis dubia Prain*	Fumariaceae	Leaf	Methanol	‐	In vitro	*T.b.b*	>10 μg/ml	>10 μg/ml	Australia	(Wangchuk, 2014)
*Meconopsis simplicifolia (D. Don) Walp*.	Papaveraceae	Leaf	Methanol	‐	In vitro	*T.b.b*	>10 μg/ml	>10 μg/ml	Australia	(Wangchuk, 2014)
*Pleurospermum amabile W. G. Craib & W.W. Sm*.	Umbellifereae	Leaf	DCM	‐	In vitro	*T.b.b*	14.83 μg/ml	>25 μg/ml	Australia	(Wangchuk, 2014)

*Note*: Their EC50 values are known.

Many plants worldwide serve as a potential source of bioactive compounds against trypanosomiases. We encountered 77 chemically defined natural molecules reported in the literature, which have been evaluated for anti‐trypanosomal activity. Fifty‐nine were from Africa, 11 from Asia, 3 from Europe and 4 from Latin America. The active compounds, isolated and identified, belong to the classes of alkaloids, triterpenoids, lactones (Kohno, et al., 2010), quinoids, flavonoids, steroids, lipids, iridoids, oxygen heterocycles, benzenoids, lignans, proteids, coumarins, phenylpropanoids and peptides. The most active compounds with EC50 of <20 µg/ml are abruquinones, letestuianin, 22‐hydroxyclerosterol, 7,15‐dihydroxy‐7,15‐deoxo nimbin, cassythine, polyacetylenes (MS‐1, MS‐2 and MS‐4), Putranoside A, kolavenol, triterpenoid (3β,13β‐dihydroxy‐urs‐11‐en‐28‐oic acid), lucidamine, oleanolic acid, phytol, betulinic acid, β‐sitosterol, citronellal, clerodane, saringosterol, 24‐hydroperoxy‐24‐vinylcholesterol, melicopicine, skimmianine, α‐amyrin, punicalagin, cedrelone, vernogui‐nosterol and diacetylvernoguinosterol, cynaropicrin, Schkuhrin I and II, saropeptide, oregonin, hirsutanone, curlone, isoiridogermanal, mahanimbine, murrayafoline, girinimbine, vanicoside E, (+)‐ketopinoresinol, isorhamnetin, cardamomin, onopordopicrin, juncunol, miltirone, isoobtusi‐lactone A, canthin‐6‐one, thus, are promising leads for drug development. Abruquinone K, L, A and D, artemisinin, MS‐2, MS‐4, dioncophylline E, dihydrochelerythrine, clerodane, Schkuhrin I, cynaropicrin, waltheriones L and vanicoside E showed inhibitory activity below 1 µg/ml or 1 µM.

According to the standards of the National Cancer Institute (NCI), a crude extract can be considered active for an EC50 ≤ 20 µg/ml (Cordell et al., 1993). Hence, most plant extracts (more than 50%) showed activity below 20 µg/ml. We highlighted the plant extracts that have the most activity below 1 µg/ml, which include *Kanahia laniflora, Arctium nemorosum*, *Crinum stuhlmannii subsp. Delagoense*, *Myristica fatua*, *Narcissus broussonetii var. grandiflorus*, *Salvia miltiorrhiza var. charbonnelii*, *Anthemis tinctoria subsp. australis*, *Casearia sylvestris var. lingua*, *Ranunculus sceleratus subsp. Multifidus*, *Alnus rubra f. pinnatisecta*, *Anogeissus leiocarpus*, *Coccoloba pubescens*, *Hoita macrostachya*, *Nuphar lutea subsp. Advena*, *Rhus integrifolia*. All active extracts belong to different families and are from different parts of the plant. Hence, it was impossible to mention the particular plant parts or specific family.

Artemisinin is an endoperoxide sesquiterpene lactone isolated from *Artemisia annua*, one of the well‐known antiparasitic and anti‐tumoural chemotherapeutic agents (Rocha et al., 2005). The impacts of Artemisinin and its derivatives on *Trypanosoma* parasites have been investigated in in vitro and animal models. These compounds effectively inhibit the metabolism of parasites, while exhibiting limited side effects on the host (Loo et al., 2017). A large number of in vitro and in vivo studies on amastigotes, epimastigotes and trypomastigotes of *Trypanosoma* have displayed that artemisinin and its derivatives have pharmacological activities in controlling the parasites and have shown significant impact against protozoans such as *T. brucei rhodesiense*, *T. brucei brucei* and *T. cruzi* (Loo et al., 2017).

### 3.9 Critical assessment of the literature information embodied in the present study

Africa, Asia and the Middle East flore provide many promising plants, but further in vivo studies are required to confirm their application as anti‐trypanosomal agents. It is worth noting that before in vivo studies, the in vitro biological activity should be accompanied by cytotoxicity studies against mammalian cells, followed by pharmacokinetic studies. On the other hand, some literature was not entered into this systematic review based on mesh terms. In West Africa and South America, *Trypanosoma vivax* is at the helm of the majority of trypanosome infections in cattle and other ruminants. This pathogen is not well established in laboratory animals, and investigation into pathogenic isolates has been restricted by the difficulty of its in vitro establishment. In this study, very few compounds were screened against *Trypanosoma vivax* (Isoun and Isoun, 1974' Cortez et al., 2006).

## 4 CONCLUSIONS

Many plants worldwide serve as a potential source of bioactive compounds against trypanosomiases. Africa, Asia and the Middle East flore provide many promising plants, but further in vivo studies are required to confirm their application as anti‐trypanosomal agents. At the same time, the isolation of the bioactive compounds in their pure form should be pursued. Further vital investigations, including clarification of their mode of action, assessment of the efficacy of several bioactive compounds and their toxicity profile, need to be carried out.

## AUTHOR CONTRIBUTIONS

Shahin Nekoui: Methodology; writing – review & editing. Faham Khamesipour: Investigation; supervision; validation; writing – original draft; writing – review & editing. Pardis Mohammadi Pour: Methodology; writing – review & editing.

## CONFLICT OF INTEREST

The authors declare that they have no competing interests.

## FUNDING

No funding was received.

## ETHICS STATEMENT

An ethics statement is not applicable because this study is based exclusively on published literature.

### PEER REVIEW

I would not like my name to appear with my report on Publons https://publons.com/publon/10.1002/vms3.912

## Data Availability

Data sharing is not applicable to this article as no new data were created or analysed in this study.

## References

[vms3912-bib-0001] Ahmad Khan, M. S. , & Ahmad, I. (2019). Chapter 1 – Herbal medicine: Current trends and future prospects. In M. S. Ahmad Khan , I. Ahmad , & D. Chattopadhyay (Eds.), New look to phytomedicine (pp. 3–13). Academic Press. 10.1016/B978-0-12-814619-4.00001-X

[vms3912-bib-0002] Al‐Musayeib, N. M. , Mothana, R. A. , Matheeussen, A. , Cos, P. , & Maes, L. (2012). In vitro antiplasmodial, antileishmanial and antitrypanosomal activities of selected medicinal plants used in the traditional Arabian Peninsular region. BMC Complementary and Alternative Medicine, 12, 49. 10.1186/1472-6882-12-49 22520595 PMC3493369

[vms3912-bib-0003] Al‐Musayeib, N. M. , Mothana, R. A. , Al‐Massarani, S. , Matheeussen, A. , Cos, P. , & Maes, L. (2012). Study of the in vitro antiplasmodial, antileishmanial and antitrypanosomal activities of medicinal plants from Saudi Arabia. Molecules (Basel, Switzerland), 17(10), 11379–11390. 10.3390/molecules171011379 PMC6268159 23011279

[vms3912-bib-0004] Atawodi, S. E. , Ameh, D. A. , Ibrahim, S. , Andrew, J. N. , Nzelibe, H. C. , Onyike, E. O. , Anigo, K. M. , Abu, E. A. , James, D. B. , Njoku, G. C. , & Sallau, A. B. (2002). Indigenous knowledge system for treatment of trypanosomiasis in Kaduna state of Nigeria. Journal of Ethnopharmacology, 79(2), 279–282. 10.1016/s0378-8741(01)00351-8 11801393

[vms3912-bib-0005] Atawodi, S. E. (2005). Comparative in vitro trypanocidal activities of petroleum ether, chloroform, methanol and aqueous extracts of some Nigerian savannah plants. African Journal of Biotechnology, 4(2), 177–182.

[vms3912-bib-0006] Bawm, S. (2010). Studies on antitrypanosomal activity of medicinal plants (p. 98). Japan: Hokkaido University.

[vms3912-bib-0007] Bero, J. , Beaufay, C. , Hannaert, V. , Hérent, M. F. , Michels, P. A. , & Quetin‐Leclercq, J. (2013). Antitrypanosomal compounds from the essential oil and extracts of Keetia leucantha leaves with inhibitor activity on *Trypanosoma brucei* glyceraldehyde‐3‐phosphate dehydrogenase. Phytomedicine: International Journal of Phytotherapy and Phytopharmacology, 20(3–4), 270–274. 10.1016/j.phymed.2012.10.010 23312849

[vms3912-bib-0008] de Bittencourt, N. L. R. , Ueda‐Nakamura, T. , Filho, B. P. D. , & Nakamura, C. V. (2011). Antitrypanosomal activity of a semi‐purified subfraction rich in Labdane Sesquiterpenes, obtained from flowers of *Anthemis tinctoria* , against *Trypanosoma cruzi*. Pharmacology & Pharmacy, 02(02), 47. 10.4236/pp.2011.22006

[vms3912-bib-0009] Bringmann, G. , Dreyer, M. , Faber, J. H. , Dalsgaard, P. W. , Staerk, D. , Jaroszewski, J. W. , Ndangalasi, H. , Mbago, F. , Brun, R. , & Christensen, S. B. (2004). Ancistrotanzanine C and related 5,1'‐ and 7,3'‐coupled naphthylisoquinoline alkaloids from *Ancistrocladus tanzaniensis* . Journal of Natural Products, 67(5), 743–748. 10.1021/np0340549 15165131

[vms3912-bib-0010] Bringmann, G. , Dreyer, M. , Faber, J. H. , Dalsgaard, P. W. , Staerk, D. , Jaroszewski, J. W. , Ndangalasi, H. , Mbago, F. , Brun, R. , Reichert, M. , Maksimenka, K. , & Christensen, S. B. (2003). Ancistrotanzanine A, the first 5,3'‐coupled naphthylisoquinoline alkaloid, and two further, 5,8'‐linked related compounds from the newly described species *Ancistrocladus tanzaniensis* . Journal of Natural Products, 66(9), 1159–1165. 10.1021/np030077b 14510589

[vms3912-bib-0011] Calderón, A. I. , Romero, L. I. , Ortega‐Barría, E. , Solís, P. N. , Zacchino, S. , Gimenez, A. , Pinzón, R. , Cáceres, A. , Tamayo, G. , Guerra, C. , Espinosa, A. , Correa, M. , & Gupta, M. P. (2010). Screening of Latin American plants for antiparasitic activities against malaria, Chagas disease, and leishmaniasis. Pharmaceutical Biology, 48(5), 545–553. 10.3109/13880200903193344 20645798

[vms3912-bib-0012] Charneau, S. , de Mesquita, M. L. , Bastos, I. M. , Santana, J. M. , de Paula, J. E. , Grellier, P. , & Espindola, L. S. (2016). In vitro investigation of Brazilian Cerrado plant extract activity against *Plasmodium falciparum*, *Trypanosoma cruzi* and *T. brucei gambiense* . Natural Product Research, 30(11), 1320–1326. 10.1080/14786419.2015.1055264 26222897

[vms3912-bib-0013] Cordell, G. A. , Kinghorn, A. D. , & Pezzuto, J. M. (1993). Separation, structure elucidation and bioassay of cytotoxic natural products. In: S. M. Colegate , R. J. Molyneux (Eds.), Bioactive natural products: Detection, isolation, and structure determination (pp. 198–201). Florida: CRC Press.

[vms3912-bib-0014] Cortez, A. P. , Ventura, R. M. , Rodrigues, A. C. , Batista, J. S. , Paiva, F. , Añez, N. , Machado, R. Z. , Gibson, W. C. , & Teixeira, M. M. (2006). The taxonomic and phylogenetic relationships of *Trypanosoma vivax* from South America and Africa. Parasitology, 133(Pt 2), 159–169. 10.1017/S0031182006000254 16650339

[vms3912-bib-0015] Desrivot, J. , Waikedre, J. , Cabalion, P. , Herrenknecht, C. , Bories, C. , Hocquemiller, R. , & Fournet, A. (2007). Antiparasitic activity of some New Caledonian medicinal plants. Journal of Ethnopharmacology, 112(1), 7–12. 10.1016/j.jep.2007.01.026 17329051

[vms3912-bib-0016] Deeks, E. D. (2019). Fexinidazole: First global approval. Drugs, 79(2), 215–220. 10.1007/s40265-019-1051-6 30635838

[vms3912-bib-0017] Dickie, E. A. , Giordani, F. , Gould, M. K. , Mäser, P. , Burri, C. , Mottram, J. C. , Rao, S. , & Barrett, M. P. (2020). New drugs for human African trypanosomiasis: A twenty first century success story. Tropical Medicine and Infectious Disease, 5(1), 29. 10.3390/tropicalmed5010029 PMC7157223 32092897

[vms3912-bib-0018] DiMasi, J. A. , & Paquette, C. (2004). The economics of follow‐on drug research and development: Trends in entry rates and the timing of development. PharmacoEconomics, 22(2 Suppl 2), 1–14. 10.2165/00019053-200422002-00002 15660473

[vms3912-bib-0019] Dyary, H. O. , Arifah, A. K. , Sharma, R. S. , Rasedee, A. , Mohd‐Aspollah, M. S. , Zakaria, Z. A. , Zuraini, A. , & Somchit, M. N. (2014). Antitrypanosomal screening and cytotoxic effects of selected medicinal plants. Tropical Biomedicine, 31(1), 89–96. 24862048

[vms3912-bib-0020] Dyary, H. O. , Arifah, A. K. , Sukari, M. A. , & Sharma, R. (2019). Antitrypanosomal and cytotoxic activities of botanical extracts from *Murraya koenigii* (L.) and *Alpinia mutica* Roxb. Tropical Biomedicine, 36(1), 94–102. 33597430

[vms3912-bib-0021] Ebiloma, G. U. , Igoli, J. O. , Katsoulis, E. , Donachie, A. M. , Eze, A. , Gray, A. I. , & de Koning, H. P. (2017). Bioassay‐guided isolation of active principles from Nigerian medicinal plants identifies new trypanocides with low toxicity and no cross‐resistance to diamidines and arsenicals. Journal of Ethnopharmacology, 202, 256–264. 10.1016/j.jep.2017.03.028 28336470

[vms3912-bib-0022] Elujoba, A. A. , Odeleye, O. , & Ogunyemi, C. (2005). Traditional medicine development for medical and dental primary health care delivery system in Africa. African Journal of Traditional, Complementary and Alternative Medicines, 2(1), 46–61.

[vms3912-bib-0023] Escobar, P. , Herrera, L. , Viviana, L. , Sandra, M. , Duran, C. , & Stashenko, E. (2009). Composición química y actividad anti‐tripanosomal de aceites esenciales obtenidos de Tagetes (Fam. Asteraceae), recolectados en Colombia. Revista de la Universidad Industrial de Santander. Salud, 41(3), 280–286.

[vms3912-bib-0024] Fairlamb, A. H. , & Horn, D. (2018). Melarsoprol resistance in African trypanosomiasis. Trends in Parasitology, 34(6), 481–492. 10.1016/j.pt.2018.04.002 29705579

[vms3912-bib-0025] Ferreira, M. E. , Nakayama, H. , de Arias, A. R. , Schinini, A. , de Bilbao, N. V. , Serna, E. , Lagoutte, D. , Soriano‐Agatón, F. , Poupon, E. , Hocquemiller, R. , & Fournet, A. (2007). Effects of canthin‐6‐one alkaloids from *Zanthoxylum chiloperone* on *Trypanosoma cruzi*‐infected mice. Journal of Ethnopharmacology, 109(2), 258–263. 10.1016/j.jep.2006.07.028 16949231

[vms3912-bib-0026] Floyd, M. R. (2013). High throughput screening of extracts from plants used in traditional Chinese medicine against *Trypanosoma brucei brucei* 427. PhD Thesis, Middle Tennessee State University.

[vms3912-bib-0027] Fotie, J. , Bohle, D. S. , Olivier, M. , Adelaida Gomez, M. , & Nzimiro, S. (2007). Trypanocidal and antileishmanial dihydrochelerythrine derivatives from *Garcinia lucida* . Journal of Natural Products, 70(10), 1650–1653. 10.1021/np0702281 17880175

[vms3912-bib-0028] Fournet, A. , Barrios, A. A. , & Muñoz, V. (1994). Leishmanicidal and trypanocidal activities of Bolivian medicinal plants. Journal of Ethnopharmacology, 41(1‐2), 19–37. 10.1016/0378-8741(94)90054-x 8170156

[vms3912-bib-0029] Freiburghaus, F. , Steck, A. , Pfander, H. , & Brun, R. (1998). Bioassay‐guided isolation of a diastereoisomer of kolavenol from *Entada abyssinica* active on *Trypanosoma brucei rhodesiense* . Journal of Ethnopharmacology, 61(3), 179–183. 10.1016/s0378-8741(98)00035-x 9705008

[vms3912-bib-0030] Githua, M. , & Hassanali, A. (2011). Antitrypanosomal Tetranotriterpenoids from *Toona ciliata* roots, Agriculture and Biology Journal of North America, 2(7), 1042–1047. 10.5251/abjna.2011.2.7.1042.1047

[vms3912-bib-0031] González‐Coloma, A. , Reina, M. , Sáenz, C. , Lacret, R. , Ruiz‐Mesia, L. , Arán, V. J. , Sanz, J. , & Martínez‐Díaz, R. A. (2012). Antileishmanial, antitrypanosomal, and cytotoxic screening of ethnopharmacologically selected Peruvian plants. Parasitology Research, 110(4), 1381–1392. 10.1007/s00436-011-2638-3 21922239

[vms3912-bib-0032] Gupta, R. , Gabrielsen, B. , & Ferguson, S. M. (2005). Nature's medicines: Traditional knowledge and intellectual property management. Case studies from the National Institutes of Health (NIH), USA. Current Drug Discovery Technologies, 2(4), 203–219. 10.2174/157016305775202937 16475917 PMC2739453

[vms3912-bib-0033] Hashemi, N. , Ommi, D. , Kheyri, P. , Khamesipour, F. , Setzer, W. N. , & Benchimol, M. (2021). A review study on the anti‐trichomonas activities of medicinal plants. International Journal for Parasitology. Drugs and Drug Resistance, 15, 92–104. 10.1016/j.ijpddr.2021.01.002 33610966 PMC7902805

[vms3912-bib-0034] Hassan, M. D. , Castanha, R. , & Wolfram, D. (2020). Scientometric analysis of global trypanosomiasis research: 1988–2017. Journal of Infection and Public Health, 13(4), 514–520. 10.1016/j.jiph.2019.10.006 31831393

[vms3912-bib-0035] Hata, Y. , Ebrahimi, S. N. , De Mieri, M. , Zimmermann, S. , Mokoka, T. , Naidoo, D. , Fouche, G. , Maharaj, V. , Kaiser, M. , Brun, R. , Potterat, O. , & Hamburger, M. (2014). Antitrypanosomal isoflavan quinones from *Abrus precatorius* . Fitoterapia, 93, 81–87. 10.1016/j.fitote.2013.12.015 24382449

[vms3912-bib-1001] Hata, Y. , Zimmermann, S. , Quitschau, M. , Kaiser, M. , Hamburger, M. , & Adams, M. (2011). Antiplasmodial and antitrypanosomal activity ofpyrethrins and pyrethroids. Journal of Agricultural and Food Chemistry, 59(17), 9172–9176. 10.1021/jf201776z 21786822

[vms3912-bib-0036] Hoet, S. , Pieters, L. , Muccioli, G. G. , Habib‐Jiwan, J. L. , Opperdoes, F. R. , & Quetin‐Leclercq, J. (2007). Antitrypanosomal activity of triterpenoids and sterols from the leaves of *Strychnos spinosa* and related compounds. Journal of Natural Products, 70(8), 1360–1363. 10.1021/np070038q 17637068

[vms3912-bib-0037] Ibrahim, M. A. , Mohammed, A. , Isah, M. B. , & Aliyu, A. B. (2014). Anti‐trypanosomal activity of African medicinal plants: A review update. Journal of Ethnopharmacology, 154(1), 26–54. 10.1016/j.jep.2014.04.012 24742753

[vms3912-bib-0038] Isoun, T. T. , & Isoun, M. J. (1974). In vitro cultivation of *Trypanosoma vivax* isolated from cattle. Nature, 251(5475), 513–514. 10.1038/251513a0 4421572

[vms3912-bib-0039] Jain, S. , Jacob, M. , Walker, L. , & Tekwani, B. (2016). Screening North American plant extracts in vitro against *Trypanosoma brucei* for discovery of new antitrypanosomal drug leads. BMC Complementary and Alternative Medicine, 16, 131. 10.1186/s12906-016-1122-0 27193901 PMC4870785

[vms3912-bib-0040] Kamnaing, P. , Tsopmo, A. , Tanifum, E. A. , Tchuendem, M. H. , Tane, P. , Ayafor, J. F. , Sterner, O. , Rattendi, D. , Iwu, M. M. , Schuster, B. , & Bacchi, C. (2003). Trypanocidal diarylheptanoids from *Aframomum letestuianum* . Journal of Natural Products, 66(3), 364–367. 10.1021/np020362f 12662093

[vms3912-bib-0100] Kande Betu Ku Mesu, V. , Mutombo Kalonji, W. , Bardonneau, C. , Valverde Mordt, O. , Ngolo Tete, D. , Blesson, S. , Simon, F. , Delhomme, S. , Bernhard, S. , Mahenzi Mbembo, H. , Mpia Moke, C. , Lumeya Vuvu, S. , Mudji E'kitiak, J. , Akwaso Masa, F. , Mukendi Ilunga, M. , Mpoyi Muamba Nzambi, D. , Mayala Malu, T. , Kapongo Tshilumbwa, S. , Botalema Bolengi, F. , … Tarral, A. (2021). Oral fexinidazole for stage 1 or early stage 2 African *Trypanosoma brucei* gambiense trypanosomiasis: A prospective, multicentre, open‐label, cohort study. The Lancet Global Health, 9(7), e999–e1008. 10.1016/S2214-109X(21)00208-4 34143998 PMC8220131

[vms3912-bib-0041] Kenguele, H. M. (2009). Biological evaluation of four selected African medicinal plants for their trypanocidal properties, mode of action, and chemical compounds. PhD Thesis, Howard University.

[vms3912-bib-0042] Kasilo, O. M. , Lusamba‐Dikassa, P. S. , Mwikisa Ngenda, C. , & Trapsida, J.‐M. (2010). An overview of the traditional medicine situation in the African region. The African Health Monitor, Online, 7–15.

[vms3912-bib-0043] Kpadonou Kpoviessi, B. G. , Kpoviessi, S. D. , Yayi Ladekan, E. , Gbaguidi, F. , Frédérich, M. , Moudachirou, M. , Quetin‐Leclercq, J. , Accrombessi, G. C. , & Bero, J. (2014). In vitro antitrypanosomal and antiplasmodial activities of crude extracts and essential oils of *Ocimum gratissimum* Linn from Benin and influence of vegetative stage. Journal of Ethnopharmacology, 155(3), 1417–1423. 10.1016/j.jep.2014.07.014 25058875

[vms3912-bib-0044] Kohno, S. , Kida, H. , Mizuguchi, M. , Shimada, J. , & S‐021812 Clinical Study Group . (2010). Efficacy and safety of intravenous peramivir for treatment of seasonal influenza virus infection. Antimicrobial Agents and Chemotherapy, 54(11), 4568–4574. 10.1128/AAC.00474-10 20713668 PMC2976170

[vms3912-bib-0045] Kumar, R. , Badarinath, K. , & Ravishankar, H. (2014). Evaluation of selected medicinal plants for their in vitro activity against trypanosomiasis. Evaluation, 9(9), 35–42.

[vms3912-bib-0046] Lawal, B. , Shittu, O. K. , Kabiru, A. Y. , Jigam, A. A. , Umar, M. B. , Berinyuy, E. B. , & Alozieuwa, B. U. (2015). Potential antimalarials from African natural products: A reviw. Journal of Intercultural Ethnopharmacology, 4(4), 318–343. 10.5455/jice.20150928102856 26649238 PMC4665028

[vms3912-bib-0047] Le, T. B. , Beaufay, C. , Nghiem, D. T. , Pham, T. A. , Mingeot‐Leclercq, M. P. , & Quetin‐Leclercq, J. (2019). Evaluation of the anti‐trypanosomal activity of vietnamese essential oils, with emphasis on *Curcuma longa* L. and its components. Molecules (Basel, Switzerland), 24(6), 1158. 10.3390/molecules24061158 PMC6471621 30909559

[vms3912-bib-0048] Llurba Montesino, N. , Kaiser, M. , Brun, R. , & Schmidt, T. J. (2015). Search for antiprotozoal activity in herbal medicinal preparations; new natural leads against neglected tropical diseases. Molecules (Basel, Switzerland), 20(8), 14118–14138. 10.3390/molecules200814118 PMC6332118 26248069

[vms3912-bib-0049] Loo, C. S. , Lam, N. S. , Yu, D. , Su, X. Z. , & Lu, F. (2017). Artemisinin and its derivatives in treating protozoan infections beyond malaria. Pharmacological Research, 117, 192–217. 10.1016/j.phrs.2016.11.012 27867026 PMC5316320

[vms3912-bib-0050] Martinez‐Peinado, N. , Cortes‐Serra, N. , Torras‐Claveria, L. , Pinazo, M.‐J. , Gascon, J. , Bastida, J. , & Alonso‐Padilla, J. (2020). Amaryllidaceae alkaloids with anti‐*Trypanosoma cruzi* activity. Parasites Vectors, 13, 299. 10.1186/s13071-020-04171-6 32522289 PMC7288428

[vms3912-bib-0051] Mbaya, A. W. , Aliyu, M. M. , & Ibrahim, U. I. (2009). The clinico‐pathology and mechanisms of trypanosomosis in captive and free‐living wild animals: A review. Veterinary Research Communications, 33(7), 793–809. 19340600 10.1007/s11259-009-9214-7

[vms3912-bib-0052] Mesquita, M. L. D. , Desrivot, J. , Fournet, A. , Paula, J. E. D. , Grellier, P. , & Espindola, L. S. (2005). Antileishmanial and trypanocidal activity of Brazilian Cerrado plants. Memórias do Instituto Oswaldo Cruz, 100(7), 783–787. 10.1590/S0074-02762005000700019 16419337

[vms3912-bib-0053] Mokoka, T. A. , Xolani, P. K. , Zimmermann, S. , Hata, Y. , Adams, M. , Kaiser, M. , Moodley, N. , Maharaj, V. , Koorbanally, N. A. , Hamburger, M. , Brun, R. , & Fouche, G. (2013). Antiprotozoal screening of 60 South African plants, and the identification of the antitrypanosomal germacranolides schkuhrin I and II. Planta Medica, 79(14), 1380–1384. 10.1055/s-0033-1350691 23929246

[vms3912-bib-0054] Molina‐Garza, Z. J. , Bazaldúa‐Rodríguez, A. F. , Quintanilla‐Licea, R. , & Galaviz‐Silva, L. (2014). Anti‐Trypanosoma cruzi activity of 10 medicinal plants used in northeast Mexico. Acta Tropica, 136, 14–18. 10.1016/j.actatropica.2014.04.006 24742906

[vms3912-bib-0055] Muñoz, O. M. , Maya, J. D. , Ferreira, J. , Christen, P. , San Martin, J. , López‐Muñoz, R. , Morello, A. , & Kemmerling, U. (2013). Medicinal plants of Chile: Evaluation of their anti‐*Trypanosoma cruzi* activity. Zeitschrift fur Naturforschung C, 68(5–6), 198–202. 23923616

[vms3912-bib-0056] Mwangi, E. S. K. , Keriko, J. M. , Machocho, A. K. , Wanyonyi, A. W. , Malebo, H. M. , Chhabra, S. C. , Mwangi, E. S. , Keriko, J. M. , Machocho, A. K. , Wanyonyi, A. W. , Malebo, H. M. , Chhabra, S. C. , Mwangi, E. S. K. , & Tarus, P. K. (2010). Antiprotozoal activity and cytotoxicity of metabolites from leaves of *Teclea trichocarpa* . Journal of Medicinal Plants Research, 4(9), 726–731.

[vms3912-bib-0057] Newman, D. J. , & Cragg, G. M. (2016). Natural products as sources of new drugs from 1981 to 2014. Journal of Natural Products, 79(3), 629–661. 10.1021/acs.jnatprod.5b01055 26852623

[vms3912-bib-0058] Newman, D. J. , Cragg, G. M. , & Snader, K. M. (2003). Natural products as sources of new drugs over the period 1981–2002. Journal of Natural Products, 66(7), 1022–1037. 10.1021/np030096l 12880330

[vms3912-bib-0059] Nezaratizade, S. , Hashemi, N. , Ommi, D. , Orhan, I. E. , & Khamesipour, F. (2021). A systematic review of anti‐*Entamoeba histolytica* activity of medicinal plants published in the last 20 years. Parasitology, 148(6), 672–684. 10.1017/S0031182021000172 33536098 PMC11010214

[vms3912-bib-0060] Nganso, Y. O. , Ngantchou, I. E. , Nkwenoua, E. , Nyasse, B. , Denier, C. , Hannert, V. , & Schneider, B. (2011). Antitrypanosomal and cytotoxic activities of 22‐Hydroxyclerosterol, a new sterol from *Allexis cauliflora* (Violaceae). Scientia Pharmaceutica, 79(1), 137–144. 10.3797/scipharm.1012-10 21617778 PMC3097502

[vms3912-bib-0061] Nibret, E. , Sporer, F. , Asres, K. , & Wink, M. (2009). Antitrypanosomal and cytotoxic activities of pyrrolizidine alkaloid‐producing plants of Ethiopia. The Journal of Pharmacy and Pharmacology, 61(6), 801–808. 10.1211/jpp/61.06.0014 19505372

[vms3912-bib-0062] Nok, A. J. (2002). Azaanthraquinone inhibits respiration and in vitro growth of long slender bloodstream forms of *Trypanosoma congolense* . Cell Biochemistry and Function, 20(3), 205–212. 10.1002/cbf.948 12125096

[vms3912-bib-0063] Ntie‐Kang, F. , Lifongo, L. L. , Mbaze, L. M. , Ekwelle, N. , Owono Owono, L. C. , Megnassan, E. , Judson, P. N. , Sippl, W. , & Efange, S. M. (2013). Cameroonian medicinal plants: A bioactivity versus ethnobotanical survey and chemotaxonomic classification. BMC Complementary and Alternative Medicine, 13, 147. 10.1186/1472-6882-13-147 23802859 PMC3703288

[vms3912-bib-0064] Nunes, F. O. , de Almeida, J. M. , Ferreira, A. , da Cruz, L. A. , Jacob, C. , Garcez, W. S. , & Garcez, F. R. (2020). Antitrypanosomal butanolides from *Aiouea trinervis* . EXCLI Journal, 19, 323–333. 10.17179/excli2020-1088 32327956 PMC7174576

[vms3912-bib-0065] Nweze, N. E. (2012). In vitro anti‐trypanosomal activity of *Morinda lucida* leaves. African Journal of Biotechnology, 11(7), 1812–1817. 10.5897/AJB11.862

[vms3912-bib-0066] Nweze, N. E. , Anene, B. M. , & Asuzu, I. U. (2011). Investigation of the antitrypanosomal activity of *Buchholzia coriacea* seed extract against a field strain of *Trypanosoma congolense* . African Journal of Traditional, Complementary, and Alternative Medicines: AJTCAM, 8(5 Suppl), 175–180. 10.4314/ajtcam.v8i5S.23 22754072 PMC3252706

[vms3912-bib-0067] Obah, A. , Lawal, M. , & Malann, Y. (2013). Anti‐*Trypanosoma* activity of the ethanolic leaf extract of *Senna occidentalis* (Fabaceae) against *T. brucei brucei* infected mice. International Journal of Basic and Applied Sciences, 2(1), 32–37.

[vms3912-bib-0068] Odhiambo, J. A. , Lukhoba, C. W. , & Dossaji, S. F. (2011). Evaluation of herbs as potential drugs/medicines. African Journal of Traditional, Complementary, and Alternative Medicines: AJTCAM, 8(5 Suppl), 144–151. 10.4314/ajtcam.v8i5S.20 PMC3252725 22754068

[vms3912-bib-0069] Oliveira, M. , Sales Junior, P. A. , Rodrigues, M. J. , DellaGreca, M. , Barreira, L. , Murta, S. M. , Romanha, A. J. , & Custódio, L. (2016). Unlocking the in vitro anti‐*Trypanosoma cruzi* activity of halophyte plants from the southern Portugal. Asian Pacific Journal of Tropical Medicine, 9(8), 735–741. 10.1016/j.apjtm.2016.06.015 27569881

[vms3912-bib-0070] Osório, A. L. , Madruga, C. R. , Desquesnes, M. , Soares, C. O. , Ribeiro, L. R. , & Costa, S. C. (2008). Trypanosoma (Duttonella) vivax: Its biology, epidemiology, pathogenesis, and introduction in the New World – A review. Memorias do Instituto Oswaldo Cruz, 103(1), 1–13. 10.1590/s0074-02762008000100001 18368231

[vms3912-bib-0071] Page, M. J. , McKenzie, J. E. , Bossuyt, P. M. , Boutron, I. , Hoffmann, T. C. , Mulrow, C. D. , Shamseer, L. , Tetzlaff, J. M. , Akl, E. A. , Brennan, S. E. , Chou, R. , Glanville, J. , Grimshaw, J. M. , Hróbjartsson, A. , Lalu, M. M. , Li, T. , Loder, E. W. , Mayo‐Wilson, E. , McDonald, S. , … Moher, D. (2021). The PRISMA 2020 statement: An updated guideline for reporting systematic reviews. BMJ (Clinical Research Ed.), 372, n71. 10.1136/bmj.n71 PMC8005924 33782057

[vms3912-bib-0072] Pathiranage, A. L. , Stubblefield, J. M. , Zhou, X. , Miao, J. , Newsome, A. L. , & Dunlap, N. (2016). Antitrypanosomal activity of iridals from Iris domestica, Phytochemistry Letters, 18, 44–50. 10.1016/j.phytol.2016.08.025

[vms3912-bib-0073] Pan American Health Organization (PAHO); World Health Organization. Epidemiological update: Neurological syndrome, congenital anomalies, and Zika virus infection. (2016). Jan 17 [cited 2018 Sep 19]. https://www.paho.org/hq/dmdocuments/2016/2016‐jan‐17‐cha‐epi‐update‐zika‐virus.pdf

[vms3912-bib-0074] Patwardhan, B. (2005). Ethnopharmacology and drug discovery. Journal of Ethnopharmacology, 100(1–2), 50–52. 10.1016/j.jep.2005.06.006 16023811

[vms3912-bib-0099] Pan American Health Organization (PAHO) ; World Health Organization. Epidemiological update: Neurological syndrome, congenital anomalies, and Zika virus infection. 2016 [cited 2018 Sep 19]. https://www.paho.org/hq/dmdocuments/2016/2016‐jan‐17‐cha‐epi‐update‐zika‐virus.pdf

[vms3912-bib-0075] Pink, R. , Hudson, A. , Mouriès, M. A. , & Bendig, M. (2005). Opportunities and challenges in antiparasitic drug discovery. Nature Reviews. Drug Discovery, 4(9), 727–740. 10.1038/nrd1824 16138106

[vms3912-bib-0076] Priotto, G. , Kasparian, S. , Mutombo, W. , Ngouama, D. , Ghorashian, S. , Arnold, U. , Ghabri, S. , Baudin, E. , Buard, V. , Kazadi‐Kyanza, S. , Ilunga, M. , Mutangala, W. , Pohlig, G. , Schmid, C. , Karunakara, U. , Torreele, E. , & Kande, V. (2009). Nifurtimox‐eflornithine combination therapy for second‐stage African *Trypanosoma brucei gambiense* trypanosomiasis: A multicentre, randomised, phase III, non‐inferiority trial. Lancet (London, England), 374(9683), 56–64. 10.1016/S0140-6736(09)61117-X 19559476

[vms3912-bib-0077] Raheem, D. J. , Tawfike, A. F. , Abdelmohsen, U. R. , Edrada‐Ebel, R. , & Fitzsimmons‐Thoss, V. (2019). Application of metabolomics and molecular networking in investigating the chemical profile and antitrypanosomal activity of British bluebells (*Hyacinthoides non‐scripta*). Scientific Reports, 9(1), 2547. 10.1038/s41598-019-38940-w 30796274 PMC6385288

[vms3912-bib-0078] Regueira‐Neto, M. , Tintino, S. R. , Rolón, M. , Coronal, C. , Vega, M. C. , de Queiroz Balbino, V. , & de Melo Coutinho, H. D. (2018). Antitrypanosomal, antileishmanial and cytotoxic activities of Brazilian red propolis and plant resin of *Dalbergia ecastaphyllum* (L) Taub. Food and chemical Toxicology: An International Journal Published for the British Industrial Biological Research Association, 119, 215–221. 10.1016/j.fct.2018.04.029 29665415

[vms3912-bib-0079] Rocha, L. G. , Almeida, J. R. , Macêdo, R. O. , & Barbosa‐Filho, J. M. (2005). A review of natural products with antileishmanial activity. Phytomedicine: International Journal of Phytotherapy and Phytopharmacology, 12(6‐7), 514–535. 10.1016/j.phymed.2003.10.006 16008131

[vms3912-bib-0080] Schinella, G. R. , Tournier, H. A. , Prieto, J. M. , Ríos, J. L. , Buschiazzo, H. , & Zaidenberg, A. (2002). Inhibition of *Trypanosoma cruzi* growth by medical plant extracts. Fitoterapia, 73(7‐8), 569–575. 10.1016/s0367-326x(02)00246-0 12490214

[vms3912-bib-0081] Senn, M. , Gunzenhauser, S. , Brun, R. , & Séquin, U. (2007). Antiprotozoal polyacetylenes from the Tanzanian medicinal plant *Cussonia zimmermannii* . Journal of Natural Products, 70(10), 1565–1569. 10.1021/np0702133 17922552

[vms3912-bib-0082] Sepúlveda‐Robles, O. , Espinoza‐Gutiérrez, B. , Gomez‐Verjan, J. C. , Guzmán‐Gutiérrez, S. L. , De Ita, M. , Silva‐Miranda, M. , Espitia‐Pinzón, C. I. , Fernández‐Ramírez, F. , Herrera‐Salazar, A. , Mata‐Rocha, M. , Ortega‐Hernández, A. , & Reyes‐Chilpa, R. (2019). Trypanocidal and toxicological assessment in vitro and in silico of three sesquiterpene lactones from Asteraceae plant species. Food and Chemical Toxicology: An International Journal Published for the British Industrial Biological Research Association, 125, 55–61. 10.1016/j.fct.2018.12.023 30572063

[vms3912-bib-0083] Shaba, P. O. , & Rao, J. R. (2011). In vitro trypanocidal activity of methanolic extracts of *Quercus borealis* leaves and Zingiber officinale roots against *Trypanosoma evansi* . Greener Journal of Agricultural Sciences, 1, 41–47.

[vms3912-bib-0084] Shuaibu, M. N. , Wuyep, P. T. , Yanagi, T. , Hirayama, K. , Ichinose, A. , Tanaka, T. , & Kouno, I. (2008). Trypanocidal activity of extracts and compounds from the stem bark of *Anogeissus leiocarpus* and *Terminalia avicennoides* . Parasitology Research, 102(4), 697–703. 10.1007/s00436-007-0815-1 18066599

[vms3912-bib-0085] Simarro, P. P. , Cecchi, G. , Franco, J. R. , Paone, M. , Diarra, A. , Ruiz‐Postigo, J. A. , Fèvre, E. M. , Mattioli, R. C. , & Jannin, J. G. (2012). Estimating and mapping the population at risk of sleeping sickness. PLoS Neglected Tropical Diseases, 6(10), e1859. 10.1371/journal.pntd.0001859 23145192 PMC3493382

[vms3912-bib-0086] Simoben, C. V. , Ntie‐Kang, F. , Akone, S. H. , & Sippl, W. (2018). Compounds from african medicinal plants with activities against selected parasitic diseases: Schistosomiasis, trypanosomiasis and leishmaniasis. Natural Products and Bioprospecting, 8(3), 151–169. 10.1007/s13659-018-0165-y 29744736 PMC5971035

[vms3912-bib-0087] Slusarczyk, S. , Zimmermann, S. , Kaiser, M. , Matkowski, A. , Hamburger, M. , & Adams, M. (2011). Antiplasmodial and antitrypanosomal activity of tanshinone‐type diterpenoids from *Salvia miltiorrhiza* . Planta Medica, 77(14), 1594–1596. 10.1055/s-0030-1270933 21412700

[vms3912-bib-0088] Tajbakhsh, E. , Khamesipour, A. , Hosseini, S. R. , Kosari, N. , Shantiae, S. , & Khamesipour, F. (2021a). The effects of medicinal herbs and marine natural products on wound healing of cutaneous leishmaniasis: A systematic review. Microbial Pathogenesis, 161(Pt A), 105235. 10.1016/j.micpath.2021.105235 34648927

[vms3912-bib-0089] Tajbakhsh, E. , Kwenti, T. E. , Kheyri, P. , Nezaratizade, S. , Lindsay, D. S. , & Khamesipour, F. (2021b). Antiplasmodial, antimalarial activities and toxicity of African medicinal plants: A systematic review of literature. Malaria Journal, 20, 349 10.1186/s12936-021-03866-0 34433465 PMC8390284

[vms3912-bib-0090] Tchinda, A. T. , Tsopmo, A. , Tane, P. , Ayafor, J. F. , Connolly, J. D. , & Sterner, O. (2002). Vernoguinosterol and vernoguinoside, trypanocidal stigmastane derivatives from *Vernonia guineensis* (Asteraceae). Phytochemistry, 59(4), 371–374. 10.1016/s0031-9422(01)00448-4 11830150

[vms3912-bib-0091] Tung, N. H. , Suzuki, M. , Uto, T. , Morinaga, O. , Kwofie, K. D. , Ammah, N. , Koram, K. A. , Aboagye, F. , Edoh, D. , Yamashita, T. , Yamaguchi, Y. , Setsu, T. , Yamaoka, S. , Ohta, N. , & Shoyama, Y. (2014). Anti‐trypanosomal activity of diarylheptanoids isolated from the bark of *Alnus japonica* . The American Journal of Chinese Medicine, 42(5), 1245–1260. 10.1142/S0192415X14500785 25178281

[vms3912-bib-0092] Wangchuk, P. (2014). Phytochemical analysis, bioassays and the identification of drug lead compounds from seven Bhutanese medicinal plants. Australia: University of Wollongong.

[vms3912-bib-0093] Welburn, S. C. , Maudlin, I. , & Simarro, P. P. (2009). Controlling sleeping sickness – A review. Parasitology, 136(14), 1943–1949. 10.1017/S0031182009006416 19691861

[vms3912-bib-0094] W. H. O. (2015) Chagas disease (*American trypanosomiasis*) 340. Available at: https://www.who.int/news‐room/fact‐sheets/detail/chagas‐disease‐(american‐trypanosomiasis) (Accessed: 13 August 2020)

[vms3912-bib-0095] W. H. O. (2018) Human African trypanosomiasis (sleeping sickness). Available at: https://www.who.int/health‐topics/human‐african‐trypanosomiasis#tab=tab_1 (Accessed: 13 August 2020)

[vms3912-bib-0096] Wilkinson, S. R. , & Kelly, J. M. (2009). Trypanocidal drugs: Mechanisms, resistance and new targets. Expert Reviews in Molecular Medicine, 11, e31. 10.1017/S1462399409001252 19863838

[vms3912-bib-0097] Xiao, H. , Rao Ravu, R. , Tekwani, B. L. , Li, W. , Liu, W. B. , Jacob, M. R. , Khan, S. I. , Cai, X. , Peng, C. Y. , Khan, I. A. , Li, X. C. , & Wang, W. (2017). Biological evaluation of phytoconstituents from Polygonum hydropiper. Natural Product Research, 31(17), 2053–2057. 10.1080/14786419.2016.1269094 28000515

[vms3912-bib-0098] Zimmermann, S. , Thomi, S. , Kaiser, M. , Hamburger, M. , & Adams, M. (2012). Screening and HPLC‐based activity profiling for new antiprotozoal leads from European plants. Scientia Pharmaceutica, 80(1), 205–213. 10.3797/scipharm.1111-13 22396915 PMC3293357

